# Oleic acid-induced metastasis of KRAS/p53-mutant colorectal cancer relies on concurrent KRAS activation and IL-8 expression bypassing EGFR activation

**DOI:** 10.7150/thno.85855

**Published:** 2023-08-21

**Authors:** Chih-Jie Shen, Ren-Hao Chan, Bo-Wen Lin, Nien-Chi Li, Ying-Hsuan Huang, Wen-Chang Chang, Ben-Kuen Chen

**Affiliations:** 1 Graduate Institute of Medical Science, College of Medicine, Taipei Medical University, Taipei 110, Taiwan, ROC.; 2 Division of Colorectal Surgery, Department of Surgery, National Cheng Kung University Hospital, College of Medicine, National Cheng Kung University, Tainan 701, Taiwan, ROC.; 3 Department of Pharmacology, College of Medicine, National Cheng Kung University, Tainan 701, Taiwan, ROC.

**Keywords:** multigene mutant CRC, oleic acid, IL-8, KRAS activation, metastasis

## Abstract

**Background:** Multigene mutations in colorectal cancer (CRC), including KRAS, BRAF, and p53, afford high metastatic ability and resistance to EGFR-targeting therapy. Understanding the molecular mechanisms regulating anti-EGFR-resistant CRC metastasis can improve CRC therapy. This study aimed to investigate the effects of IL-8 and the activation of KRAS on reactive oxygen species (ROS) production and metastasis of hyperlipidemia-associated CRC harboring mutations of KRAS and p53.

**Methods:** The cytokine array analysis determined the up-expression of secreted factors, including IL-8. The clinical relevance of the relationship between IL-8 and angiopoietin-like 4 (ANGPTL4) was examined in CRC patients from National Cheng Kung University Hospital and TCGA dataset. Expressions of IL-8, ANGPTL4, NADPH oxidase 4 (NOX4), and epithelial-mesenchymal transition (EMT) markers in free fatty acids (FFAs)-treated KRAS/p53 mutant CRC cells were determined. The hyperlipidemia-triggered metastatic ability of CRC cells under treatments of antioxidants, statin, and cetuximab or knockdown of IL-8, KRAS, and EGFR was evaluated in vitro and in vivo. In addition, the effects of antioxidants and depletion of IL-8 and KRAS on the correlation between ROS production and hyperlipidemia-promoted CRC metastasis were also clarified.

**Results:** In this study, we found that free fatty acids promoted KRAS/p53-mutant but not single-mutant or non-mutant CRC cell metastasis. IL-8, the most abundant secreted factor in KRAS/p53-mutant cells, was correlated with the upregulation of NOX4 expression and ROS production under oleic acid (OA)-treated conditions. In addition, the metastasis of KRAS/p53-mutant CRC relies on the ANGPTL4/IL-8/NOX4 axis and the activation of KRAS. The antioxidants and inactivation of KRAS also inhibited OA-induced EMT and metastasis. Although KRAS mediated EGF- and OA-promoted CRC cell invasion, the inhibition of EGFR did not affect OA-induced ANGPTL4/IL-8/NOX4 axis and CRC metastasis. The high-fat diet mice fed with vitamin E and statin or in IL-8-depleted cells significantly inhibited tumor extravasation and metastatic lung growth of CRC.

**Conclusion:** The antioxidants, statins, and targeting IL-8 may provide better outcomes for treating metastatic CRC that harbors multigene mutations and anti-EGFR resistance.

## Introduction

The incidence rate of colorectal cancer (CRC) is higher for patients aged 50 years and above than for those between 20 and 49 years of age 1. Approximately 35% of CRC patients eventually develop metastasis and die in 6-20 months without treatment 2. Even if treated with chemotherapy and surgery, only 10% of patients survive longer than 5 years 3. Several genes have been identified as responsible for the increased proliferation, invasion, progression, and inhibition of apoptosis in CRC cells, including EGFR, KRAS, RAF, Notch-1, PI3KCA, and PTEN 4. Recent studies have indicated multigene mutations that impact survival in colorectal liver metastasis, including p53 (10%), KRAS (50.7%), PIK3CA (15.8%), and Smad4 (11%). Coexisting mutations in p53, KRAS, and Smad4 were associated with significantly worse overall survival and recurrence-free survival than mutations in one or none of those genes 5. In addition, identifying circulating tumor DNA using the molecular detection of APC, KRAS, and p53 gene mutations is a potential tool for the early detection of postoperative recurrence and metastases, which may be correlated with poor clinical outcomes in CRC patients 6. These results indicate that CRC metastasis and poor outcomes are highly associated with multigene mutations.

In addition to oncogenic mutations and signaling pathways, such as EGFR, TGFβ, and Wnt, in regulating CRC progression, a microenvironmental status with metabolic disorders, including chronic inflammatory bowel syndrome, type 2 diabetes, and obesity, generates a higher risk of CRC progression 7, 8. Previous studies have demonstrated that cytokines, such as IL-1, IL-6, IL-8, and TNF-α, are critical molecular mediators of cancer progression 9. Metabolic syndrome, particularly obesity, creates a tumor microenvironment containing local enrichment of adipose tissues and lipid metabolites, which is a significant risk factor for poor outcomes in various cancers, including CRC 10. It is worth noting that high lipid content was significantly associated with KRAS-mutated metastatic rectosigmoid cancers 11. In addition, our previous studies have shown that free fatty acids stimulate the production of reactive oxygen species (ROS), which leads to CRC metastasis 12. These studies suggest that fatty acids are associated with CRC metastasis. However, the correlation and underlying molecular mechanisms involved in the interplay between lipid metabolite-associated ROS and metastasis of multigene mutant CRC, including KRAS mutation, have not been elucidated.

Oxidative stress refers to an imbalance between the production and accumulation of ROS in cells and tissues 13. Obesity is associated with chronic inflammation by activating the innate immune system in adipose tissue, which promotes proinflammatory status and oxidative stress. In other words, adipose tissue has been identified as a source of proinflammatory cytokines, including TNF-α, IL-1β, and IL-6, which promote the increased generation of ROS 14. Excessive fat accumulation in obese patients also increases serum free fatty acid levels, free radical synthesis and oxidative stress 15. Silencing of the oxidant source NADPH oxidases (NOXs), especially NOX4, inhibits palmitate-stimulated ROS generation 16. Our previous studies also showed that the depletion of NOX4 inhibited oleic acid (OA)-induced ROS production in CRC cells 12. In cancer therapy, anticancer drugs have also been shown to produce oxidative stress and inflammation, accompanied by high lipid peroxidation 17, 18. Although these studies suggest a correlation between lipid content and oxidative stress, the molecular mechanism regulating ROS production in fatty acid-promoted CRC metastasis remains incompletely identified.

The challenges of CRC therapy can be caused by mutations of KRAS or BRAF, which have no response to anti-EGFR monoclonal antibodies, such as cetuximab 19. Although anticancer drugs, including FOLFOX, have been investigated in metastatic CRC, none have shown clinical efficacy in patients with KRAS-mutant tumors 20. In other words, the KRAS mutant has been recognized as a poor prognostic marker in metastatic CRC. In this study, we identified CRC containing KRAS and p53 mutations that responded to fatty acid stimulation followed by enhanced tumor metastasis. The ANGPTL4/IL-8/NOX4 axis confers fatty acid- but not EGFR signaling-promoted CRC metastasis. Our findings provide new insights to link the correlation between the ANGPTL4/IL-8/NOX4 axis and metastasis of CRC harboring KRAS and p53 mutations in hyperlipidemia.

## Materials and methods

### Cell culture

Cell lines of colorectal cancer HCT116, SW480, SW620, DLD-1, HT-29, Caco2, and Colo320dm were provided by the Research Center of Clinical Medicine, National Cheng Kung University Hospital and Bioresource Collection and Research Center (BCRC, Hsinchu City, TW). Cell lines were maintained at 37 °C in 10-cm plastic dishes containing 7 mL of culture medium. Each medium was supplemented with 10% fetal bovine serum (Invitrogen, Grand Island, NY, USA), 100 μg/mL streptomycin (Sigma-Aldrich, St Louis, MO, USA), and 100 unit/ mL penicillin (Sigma-Aldrich). SW480 and SW620 were cultured with Leibovitz's L-15 medium (Invitrogen); HCT116 and HT-29 were cultured with McCoy's 5a medium (Invitrogen); Colo320dm and DLD-1 were cultured with RPMI 1640 medium (Invitrogen). Caco2 was cultured with minimum essential culture medium (MEM; Invitrogen).

### Reagents and peptide

Salt oleic acid (sodium oleate; sc-215879) was purchased from Santa Cruz (Santa Cruz). Linoleic acid sodium salt (L8134), sodium palmitate (P9767), N-acetylcysteine (NAC; A1965), U0126 (19-147), gefitinib (SML1657), and erlotinib (SML2156) were purchased from Sigma‒Aldrich (Sigma‒Aldrich, St Louis, MO, USA). Bovine serum albumin (fatty acid-free; A7030) (Sigma‒Aldrich, St Louis, MO, USA) was conjugated with oleic acid at a 1:8 molar ratio (BSA/OA). Carboxyl H2-DCFDA (D399) and EGF (PHG0311) were purchased from Invitrogen (Invitrogen, Grand Island, NY, USA). Recombinant human ANGPTL4 (rh-ANGPTL4: 4487-AN) was purchased from R&D Systems (R&D Systems, Minneapolis, MN). CXCL8 (ab9631) was purchased from Abcam (Abcam). Cetuximab was purchased from BioVision (A1047-100; BioVision). Vitamin E (25985), hygromycin B (hydrate: 14291), reparixin (21492), and atorvastatin (10493) were purchased from Cayman (Cayman). Atorvastatin (Lipitor; VITARIS) was used for feeding mice.

### Cytokine array analysis

The conditioned medium was harvested from cultured cells without particulates, which were removed by filtration through a 0.2-μm filter. Cytokines were detected with a Proteome Profiler Human Cytokine Array Kit (ARY005B; R&D Systems). After blocking, membranes were incubated with samples and an antibody cocktail overnight at 4 °C and then incubated with streptavidin-HRP at room temperature for 30 min. The membrane was incubated with chemiluminescent detection reagents for 1 min, and the signal intensities on the membranes were detected with iBright Imaging Systems (Thermo Fisher Scientific).

### Real-time quantitative PCR

The total RNA of cells was extracted using TRIzol® (Invitrogen, Carlsbad, CA, USA). According to the manufacturer's instructions, a reverse transcriptase reaction was performed using a kit (Applied Biosystems, Foster City, CA, USA). Following cDNA synthesis, gene-specific primers were designed using NCBI primer design software. Real-time quantitative PCR was performed in triplicate using an SYBER Green MasterMix (Invitrogen) and StepOne Real-Time PCR Systems (Applied Biosystems, Carlsbad, CA, USA). Each well contained the following reaction mixture: 1 μL of cDNA, 5 μL of 2 × SensiMix Syber Green PCR reagents (Bioline, London, UK), 4 μL of RNase-free water, 0.5 μL of sense primer, and 0.5 μL of antisense primer. Universal cycling conditions were used. Relative gene expression was calculated using the comparative Ct method. All values were normalized to the housekeeping gene GAPDH. Primer sets for real-time quantitative PCR were the following: NOX1 specific primers (sense, 5'- CGC TCC CAG CAG AAG GTT GTG ATT ACC AAG G -3'; antisense, 5'- GGG GTG ACC CCA ATT CCT GCT CCA ACC A -3'), NOX4 specific primers (sense, 5'- CTC AGC GGA ATC AAT CAG CTG TG -3'; antisense, 5'- AGA GGA ACA CGA CAA TCA GCC TTA G -3'), MMP-1 specific primers (sense, 5'- ATG CAC AGC TTT CCT CCA CT -3'; antisense, 5'- TTC CCA GTC ACT TTC AGC CC -3'), MMP-3 specific primers (sense, 5'- GCA AGA CAG CAA GGC ATA GAG -3'; antisense, 5'- CCG TCA CCT CCA ATC CAA GG -3'), MMP-9 specific primers (sense, 5'- ACC TCG AAC TTT GAC AGC GAC A -3'; antisense, 5'- GAT GCC ATT CAC GTC GTC CTT A -3'), Vimentin specific primers (sense, 5'- TGG CCG ACG CCA TCA ACA CC -3'; antisense, 5'- CAC CTC GAC GCG GGC TTT GT -3'), IL-8 specific primers (sense, 5'- TTG GCA GCC TTC CTG ATT TCT -3'; antisense, 5'- TCT CAG CCC TCT TCA AAA ACT TCT C -3'), IL-6 specific primers (sense, 5'- ATG AAC TCC TTC TCC ACA AGC -3'; antisense, 5'- GTT TTC TGC CAG TGC CTC TTT G -3'), ANGPTL4 specific primers (sense, 5'- CAA GGC TCA GAA CAG CAG GA -3'; antisense, 5'- CCC CTG AGG CTG GAT TTC AA -3'), GAPDH specific primers (sense, 5'- CCA TCA CCA TCT TCC AGG AG -3'; antisense, 5'- CCT GCT TCA CCA CCT TCT TG -3'), MMP2 specific primers (sense, 5'- GCA AGT TTC CAT TCC GC -3'; antisense, 5'- GTC GTC ATC GTA GTT GGC -3'), KRAS specific primers (sense, 5'- CAG TAG ACA CAA AAC AGG CTC AG -3'; antisense, 5'- TGT CGG ATC TCT CTC ACC AAT G -3'), EGFR specific primers (sense, 5'- GCC TCC AGA GGA TGT TCA ATA A -3'; antisense, 5'- TGA GGG CAA TGA GGA CAT AAC -3'). All primers were synthesized by Genomics (Genomics, Taiwan).

### Enzyme-linked immunosorbent assay

Quantification of secretory ANGPTL4 and IL-8 in the culture medium was achieved by a DuoSet® Human Enzyme-linked immunosorbent assay (ELISA) development kit (DY3458 and DY208) according to the manufacturer's instructions (R&D Systems). Briefly, 100 μL of culture medium was incubated with precoated capture antibody at room temperature in a 96-well microplate. After washing, 100 μL of detection antibody was added for 2 h at room temperature. Then, 100 μL of the working dilution of streptavidin-HRP was added to each well for 20 min under protection from light. After washing, 100 μL of substrate solution was added for 20 min. After adding 50 μL of stop solution, a SpectraMax i3x Microplate Absorbance Reader (Molecular Devices, CA, USA) was used to quantify the optical density at a wavelength of 450 nm.

### Western blotting

Western blotting using 12% SDS‒PAGE was performed, and 30 μg of cell lysates were analyzed as previously described 12. Antibodies against human NOX4 (GTX121929) and pCHOP Ser30 (GTX55401) (GeneTex, Hsinchu, TWN); ANGPTL4 (#409800) and pFAK Tyr397 (44-624G) (Invitrogen) (BD Transduction Laboratory, Los Angeles, CA, USA); p41/42 MAPK (#9102), pAKT Ser473 (4051S) and pEGFR Y1068 (3777S) (Cell Signaling Technology, Inc., Danvers, MA, USA); GAPDH (sc-32233) and EGFR (A-10) (sc-373746) (Santa Cruz Biotechnology, Inc, Santa Cruz, CA, USA); CXCL8 (MAB208) (R&D); CHOP (A20987) (Abclonal); and NOX4 (ab109225) (Abcam) were used as the primary antibodies.

### Transfection of cells with siRNA oligonucleotides or plasmids

Transient transfection of cells with 20 nM siRNA oligonucleotides or plasmids was performed using RNAiMAX or Lipofectamine 2000 (Invitrogen) according to the manufacturer's instructions with slight modifications. The siRNA IDs were as follows: ANGPTL4 (HSS181878 and HSS181879); NOX4 (HSS121312 and HSS121313); COX2 (HSS183839); KRAS (s7939); EGFR (s564); CXCL8 (s7328 and s7327) (Invitrogen); and negative control siRNAs (D-001810-10-50) (Dharmacon, Lafayette, CO, USA). For transfection, 3.75 μL of RNAiMAX or Lipofectamine 2000 was incubated with siRNA or plasmid in 1.5 mL of Opti-MEM medium (Invitrogen) for 30 min at room temperature. Following the removal of Opti-MEM medium and replacement with 3 mL of fresh culture medium, the cells were incubated for an additional 24 h, unless stated otherwise.

### Flow cytometric analysis of reactive oxygen species

Cells were stained with 100 nM carboxyl-H2DCFDA for 30 min in PBS and then recovered in 10% FBS medium for 15 min. Cells were then trypsinized and resuspended in PBS. The DCFDA emission was measured in the green channel (FL1) on a BD Accuri™ C6 Plus (BD Biosciences, San Jose, CA, USA) flow cytometer. Ten thousand events were collected for each sample.

### Transwell migration and invasion assays

Both assays were performed using Millicell™ hanging cell culture inserts (polyethylene terephthalate (PET) membranes with 8 µm pores) (Millipore, Bedford, MA, USA). The 2 × 10^5^ cells were treated with or without 200 μM OA or 50 ng/mL EGF and then seeded in the upper chamber with a diluted Matrigel-coated membrane (Millipore) (invasion assay) or non-Matrigel-coated membrane (migration assay). After 72 h (invasion) or 24 h (migration) of incubation, the cells in the upper chamber were removed. The invaded cells at the bottom of the PET membrane were fixed with 4% paraformaldehyde and stained with 0.5% crystal violet (Sigma‒Aldrich). Crystal violet-stained cells were then solubilized with 10% acetic acid and measured at a wavelength of 595 nm using a microplate reader.

### Tumor extravasation assay in an animal model

For the lung extravasation assay, 1,1'-dioctadecyl-3,3,3',3'-tetramethylindocarbocyanine perchlorate (Dil)-labeled cells (1 × 10^6^) were resuspended in 100 μL of PBS, injected into the tail vein of 6-week-old male severe combined immunodeficiency (SCID) mice for 2 days and then sacrificed. The lungs were fixed with 4% paraformaldehyde and 30% sucrose and finally embedded in FSC 22 (#3801480) (Leica, CA, USA) prior to slicing to generate cryosections (8 μm). Immunohistochemistry (IHC) was then performed to determine the location of the vessel with an anti-CD31 antibody (ab28364) (Abcam, MA, USA). Quantification was performed by analyzing at least four sections and six fields to determine the number of tumor cells that underwent extravasation. All mice were obtained from the National Cheng Kung University (NCKU) Laboratory Animal Center (Tainan, TWN). The animal study was approved (Approval No. NCKU-IACUC-107-194 and NCKU-IACUC-109-069) by the IACUC of Laboratory Animal Center, Medical College, NCKU.

### Analysis of the pulmonary metastasis of tumors in a mouse model

Tumor cells (1 × 10^6^) were resuspended in 100 μL of Hank's balanced salt solution and then injected into the tail vein of mice. To evaluate lung metastasis, mice were sacrificed up to 12 weeks after injecting tumor cells. H&E staining of lung tissue was imaged with whole slide screening using TissueGnostics FACS-like Tissue Cytometry. The number of lung nodules that underwent cancer metastasis was quantified by analyzing the whole lung. All mice were obtained from the National Cheng Kung University (NCKU) Laboratory Animal Center (Tainan, TWN). The animal study was approved (Approval No. NCKU-IACUC-107-194 and NCKU-IACUC-109-069) by the IACUC of Laboratory Animal Center, Medical College, NCKU.

### Statistical analysis

Statistical analysis was performed using Prism 6.0 software (GraphPad Software, Inc., San Diego, CA, USA). All data are presented as the mean ± standard error of the mean (SEM) determined from individual samples (n = 3). The two-tailed Student's t test was used to compare measurable variants between two groups. Survival curves were calculated using the Kaplan‒Meier method, and differences were assessed by the log-rank test. A p value less than 0.05 was considered significant and denoted by *. P values less than 0.01 and 0.001 are denoted by ** and ***, respectively.

## Results

### The concurrent expression of IL-8 and ANGPTL4 is present in CRC tissues and OA-treated cells

**Figure 1:**
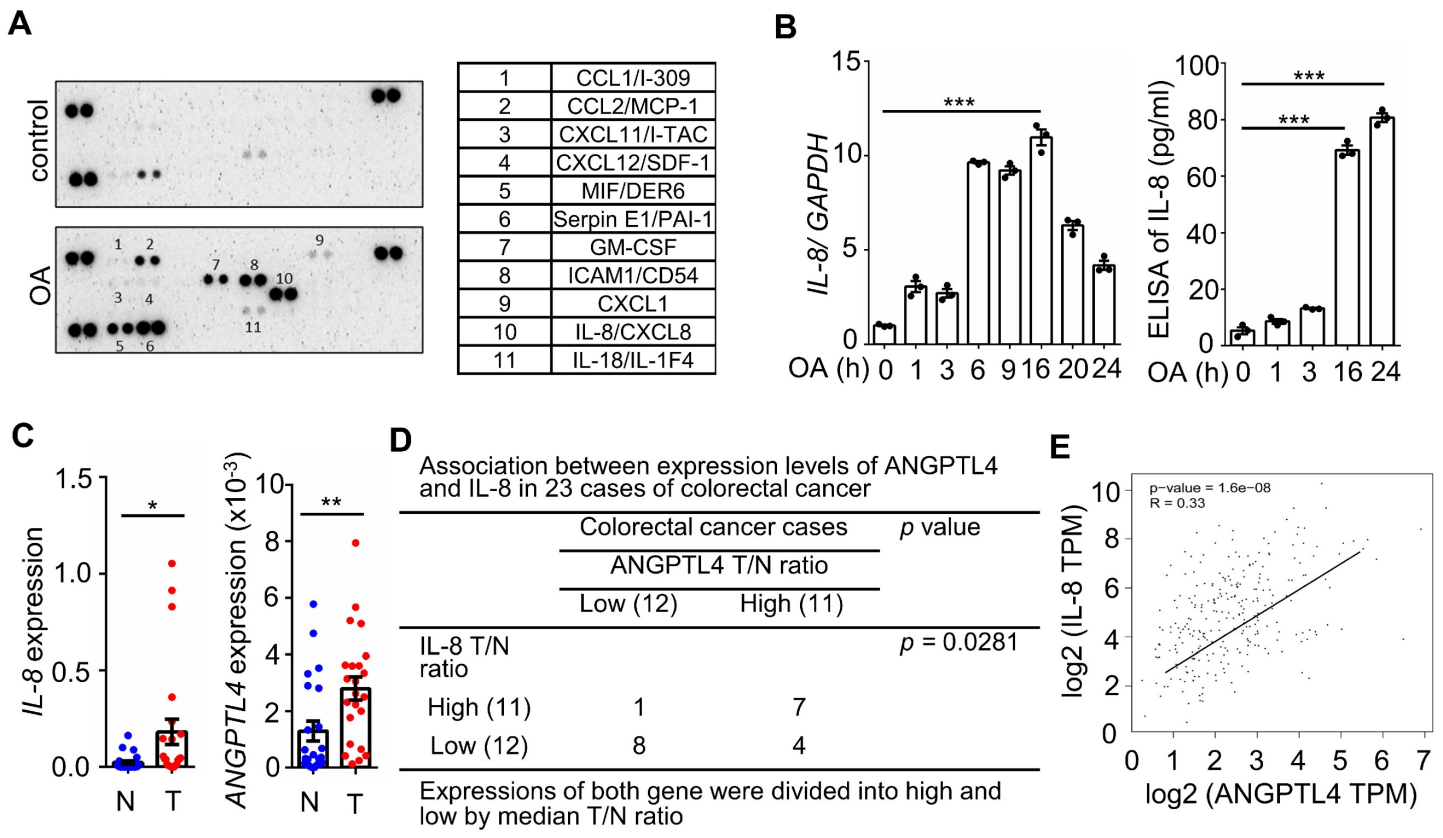
** Oleic acid induces IL-8 expression concurrently with ANGPTL4 expression in CRC tissues. (A)** The cytokine levels of conditioned medium harvested from oleic acid (OA)-treated SW480 cells were examined using the human cytokine array (R&D). The table shows the numerical values of dot blots indicating cytokine protein levels. **(B)** Real-time quantitative PCR analysis and ELISAs were performed to detect IL-8 mRNA levels and IL-8 secretion in SW480 cells treated with 200 μM OA for the indicated period. **(C-D)** Comparing of IL-8 and ANGPTL4 mRNA levels in 23 paired CRC tissues (T) and adjacent normal tissues (N) samples from National Cheng Kung University Hospital (IRB No. ER-112-111) (n = 23). The samples were analyzed by real-time quantitative PCR (C). Fisher's exact test was used for analyzing the correlation between ANGPTL4 (T/N ratio) and IL-8 (T/N ratio) (D). **(E)** Concurrent expression of ANGPTL4 and IL-8 in tumor tissues of CRC patients from the TCGA database (n = 597) was quantified (Pearson's correlation coefficient is shown in the figures; Ref: Genes Chromosomes Cancer. 2010, 49:1024-34).

**Figure 2:**
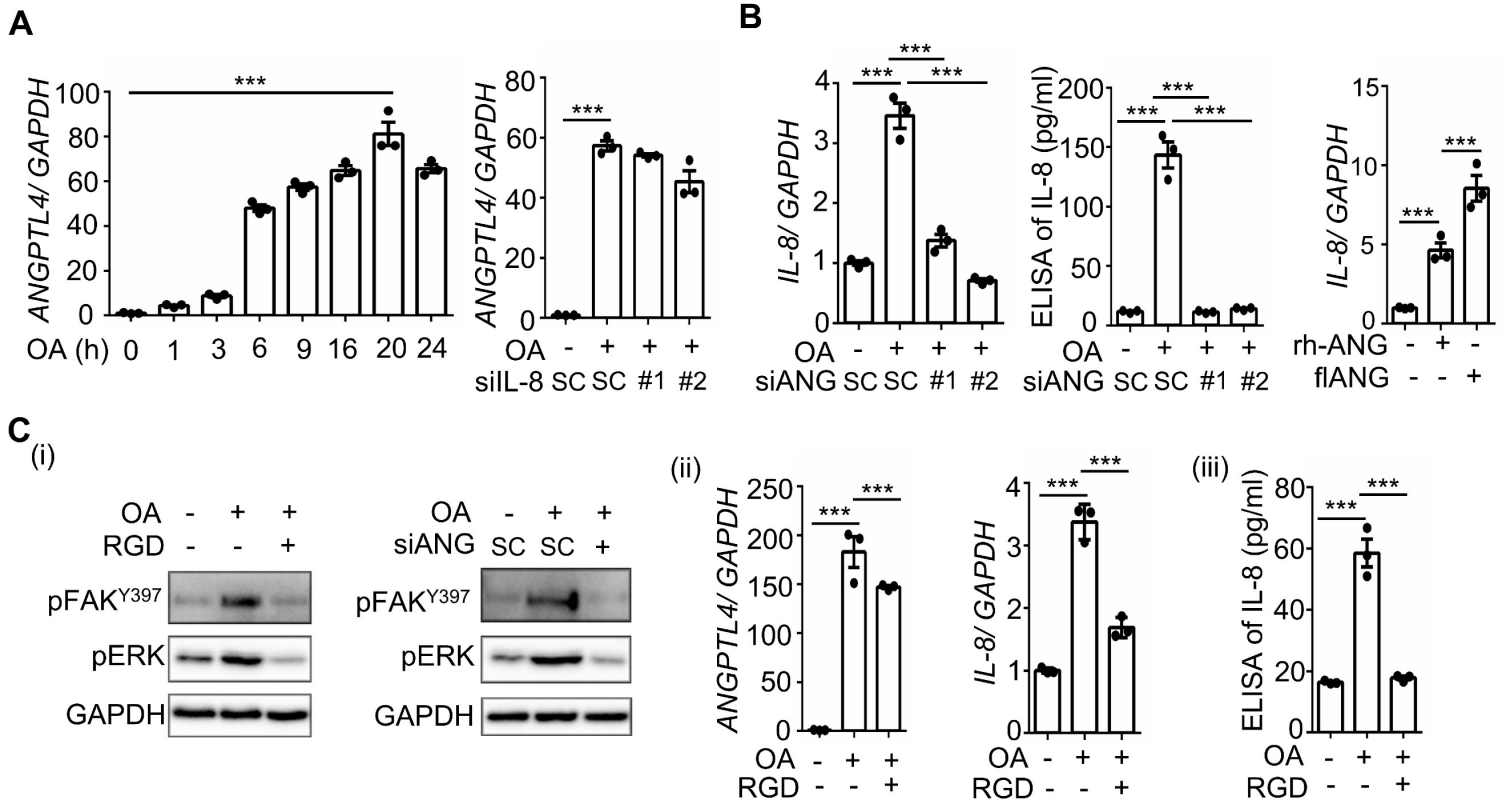
** OA-induced IL-8 expression relies on the ANGPTL4/integrin axis in CRC cells. (A-B)** Real-time quantitative PCR analysis and ELISAs were performed to examine ANGPTL4 (A) and IL-8 (B) mRNA levels and protein levels (ELISA) in SW480 cells transfected with 5 nM IL-8 siRNA, ANGPTL4 siRNA or scrambled oligonucleotides (SC) and then treated with 200 μM OA for the indicated periods or 16 h. Cells were also transfected with 0.5 μg the full-length ANGPTL4 expression vector (flANG) or treated with 100 ng/mL recombinant human ANGPTL4 (rh-ANG) for 24 h, followed by the analysis of IL-8 mRNA expression by real-time quantitative PCR (B). **(C)** Western blotting (i), real-time quantitative PCR analysis (ii), and ELISAs (iii) were performed to examine the phosphorylation of FAK and ERK; mRNA levels of ANGPTL4 and IL-8 and IL-8 protein in cells treated with 5 nM ANGPTL4 siRNA or 100 μM RGD peptide, followed by treatment with 200 μM OA for 30 min (i) and 16 h (ii and iii).

Hyperlipidemia-regulated cancer progression is associated with cytokines and chemokines 21, 22. To investigate the secreted molecules regulated by fatty acids, a human cytokine array was used to examine the expression pattern of cytokines, chemokines, growth factors, and other soluble proteins in OA-treated CRC cells. As shown in Figure 1A, the cytokine IL-8, chemokines CCL1, CCL2, CXCL1, CXCL11, and CXCL12, and growth factor GM-CSF were significantly upregulated under OA treatment. Among these secreted factors, the change in IL-8 was the most significant in OA-treated cells. We verified that OA induced the expression of IL-8 mRNA and protein in a time-dependent manner (Figure 1B). In addition, the expression of IL-8 was also higher in tumors than in surrounding normal tissues surveyed by the dataset (Figure S1). Because our previous studies revealed that OA-induced CRC metastasis was dependent on ANGPTL4 expression 12, we hypothesized that the interplay between OA-induced IL-8 and ANGPTL4 may regulate CRC metastasis. First, we analyzed the expression of IL-8 and ANGPTL4 in the tumor tissues of CRC patients. The results showed concurrently higher expression levels of IL-8 and ANGPTL4 in tumors than in the surrounding normal tissues from our clinical CRC samples of National Cheng Kung University Hospital and the TCGA dataset (Figure 1C-E). To verify whether IL-8 and ANGPTL4 expression regulate each other, knockdown of IL-8 and ANGPTL4 or overexpression of ANGPTL4 was applied in OA-treated cells. As shown in Figure 2A, the depletion of IL-8 did not affect OA-induced ANGPTL4 expression. However, OA-induced IL-8 expression was dramatically reduced by ANGPTL4 knockdown (Figure 2B). Treatment with recombinant human ANGPTL4 protein or overexpression of ANGPTL4 also significantly induced IL-8 expression (Figure 2B). On the other hand, in OA-treated cells by introduction of an inhibitor of integrin-ligand interactions, such as RGD, and depletion of ANGPTL4, inhibited not only the integrin downstream FAK and ERK signaling pathways but also IL-8 expression (Figure 2C). These results indicate that the ANGPTL4/integrin axis mediated OA-induced IL-8 expression in CRC cells.

### OA-induced IL-8 triggers NOX4 expression and ROS production

**Figure 3:**
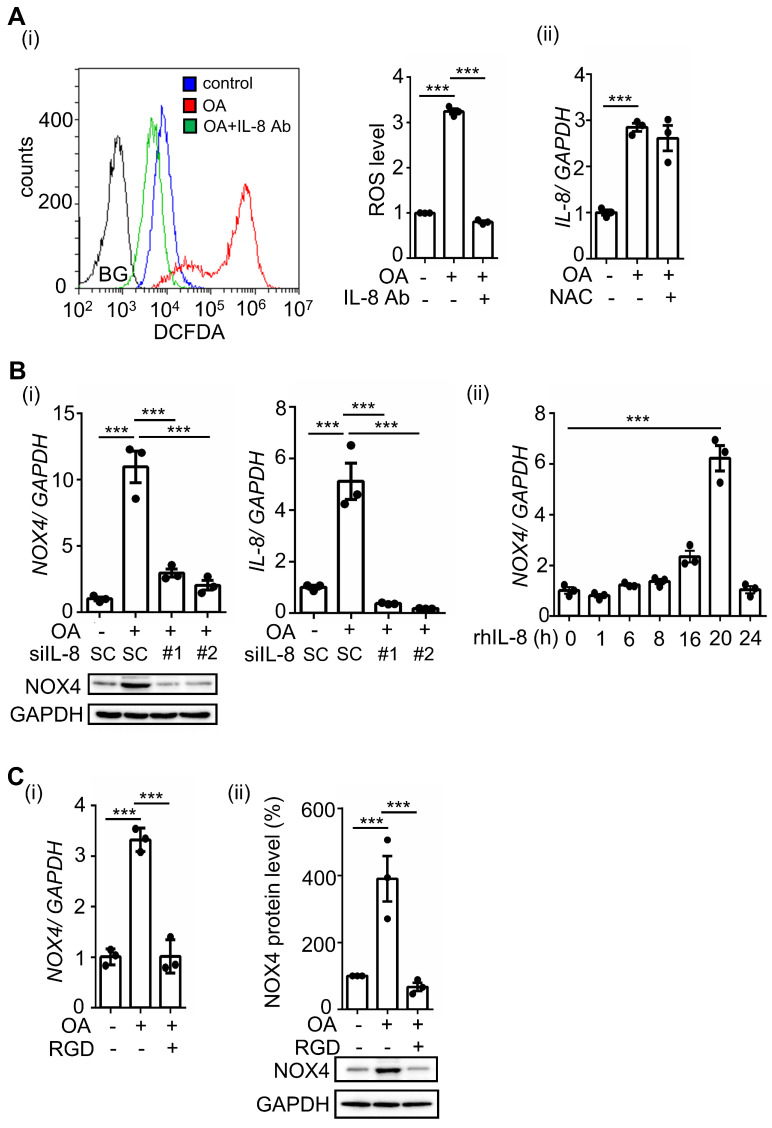
** OA-induced IL-8 regulates ROS production and NOX4 expression. (A)** SW480 cells were treated with 200 μM OA, 100 ng/mL IL-8 antibodies, and 10 mM NAC for 16 h. ROS levels (i) and IL-8 expression (ii) were detected by flow cytometry analysis with 200 nM DCFDA staining and real-time quantitative PCR analysis, respectively. BG indicates background. **(B)** Real-time quantitative PCR analysis and western blotting were performed to examine the mRNA and protein levels of IL-8 and NOX4 in SW480 cells transfected with 5 nM IL-8 siRNA or scrambled oligonucleotides (SC) and treated with 200 μM OA for 16 h (i) or 20 ng/mL recombinant human IL-8 (rh-IL-8) for various periods (ii). **(C)** Cells were also treated with 200 μM OA and 100 μM RGD for 16 h. The mRNA (i) and protein levels (ii) of NOX4 were examined using real-time quantitative PCR analysis and western blotting.

Our previous studies showed that OA-induced ANGPTL4 promotes ROS production by inducing NOX4 12. We predict that OA-induced IL-8 can also mediate ROS production. Indeed, we found that inhibition of IL-8 by treatment with IL-8-neutralizing antibodies dramatically reduced OA-induced ROS production (Figure 3A). In addition, NAC, a ROS scavenger, did not affect IL-8 expression, suggesting that ROS do not regulate IL-8 expression (Figure 3A). To further study whether IL-8-induced ROS production occurs through regulation of NOX4 expression, cells were treated with recombinant human IL-8 protein. First, the depletion of IL-8 significantly inhibited OA-induced NOX4 expression, and consistently, the introduction of recombinant human IL-8 triggered NOX4 expression (Figure 3B). These results suggest that OA-induced IL-8 expression promoted NOX4 expression and ROS production. To study whether ANGPTL4/integrin signaling mediates OA-induced NOX4 expression, the effect of RGD on NOX4 expression was examined in OA-treated cells. The results showed that RGD inhibited OA-induced NOX4 expression (Figure 3C). These results clarify that the ANGPTL4/integrin/IL-8/NOX4 signaling pathway is essential for OA-induced ROS production.

### OA-induced IL-8 expression promotes CRC metastasis

**Figure 4:**
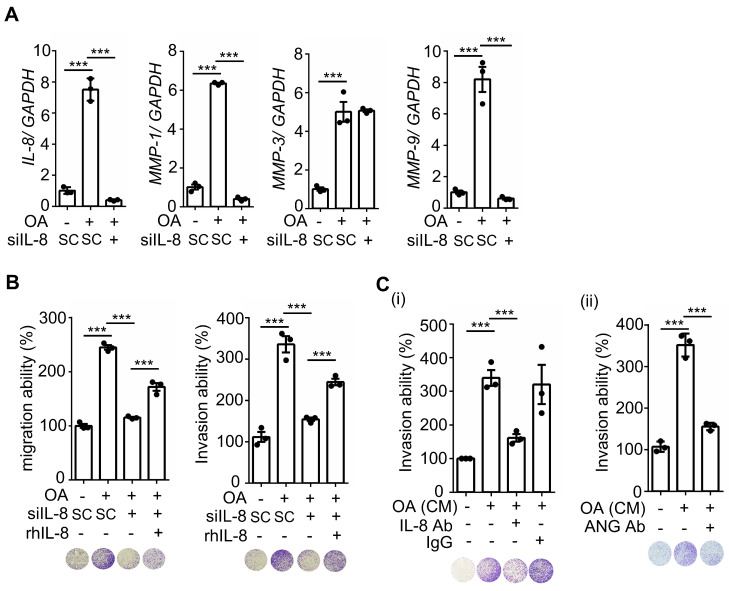
** OA-induced IL-8 enhances EMT markers and invasion and migration of CRC cells. (A)** Real-time quantitative PCR analysis was performed to examine IL-8, MMP-1, MMP-9, and MMP-3 mRNA levels in SW480 cells transfected with 5 nM IL-8 siRNA and treated with 200 μM OA for 16 h. **(B-C)** Invasion and migration assays were performed in SW480 cells transfected with 5 nM IL-8 siRNA or scrambled oligonucleotides (SC) and then treated with 200 μM OA and 20 ng/mL recombinant human IL-8 (rh-IL8) (B) or with conditioned medium harvested from OA-treated cells and anti-IL-8 and anti-ANGPTL4 antibodies (C). The penetrating cells were stained with crystal violet, imaged under a microscope, and then solubilized with 10% acetic acid. The absorbance was measured at a wavelength of 595 nm.

**Figure 5:**
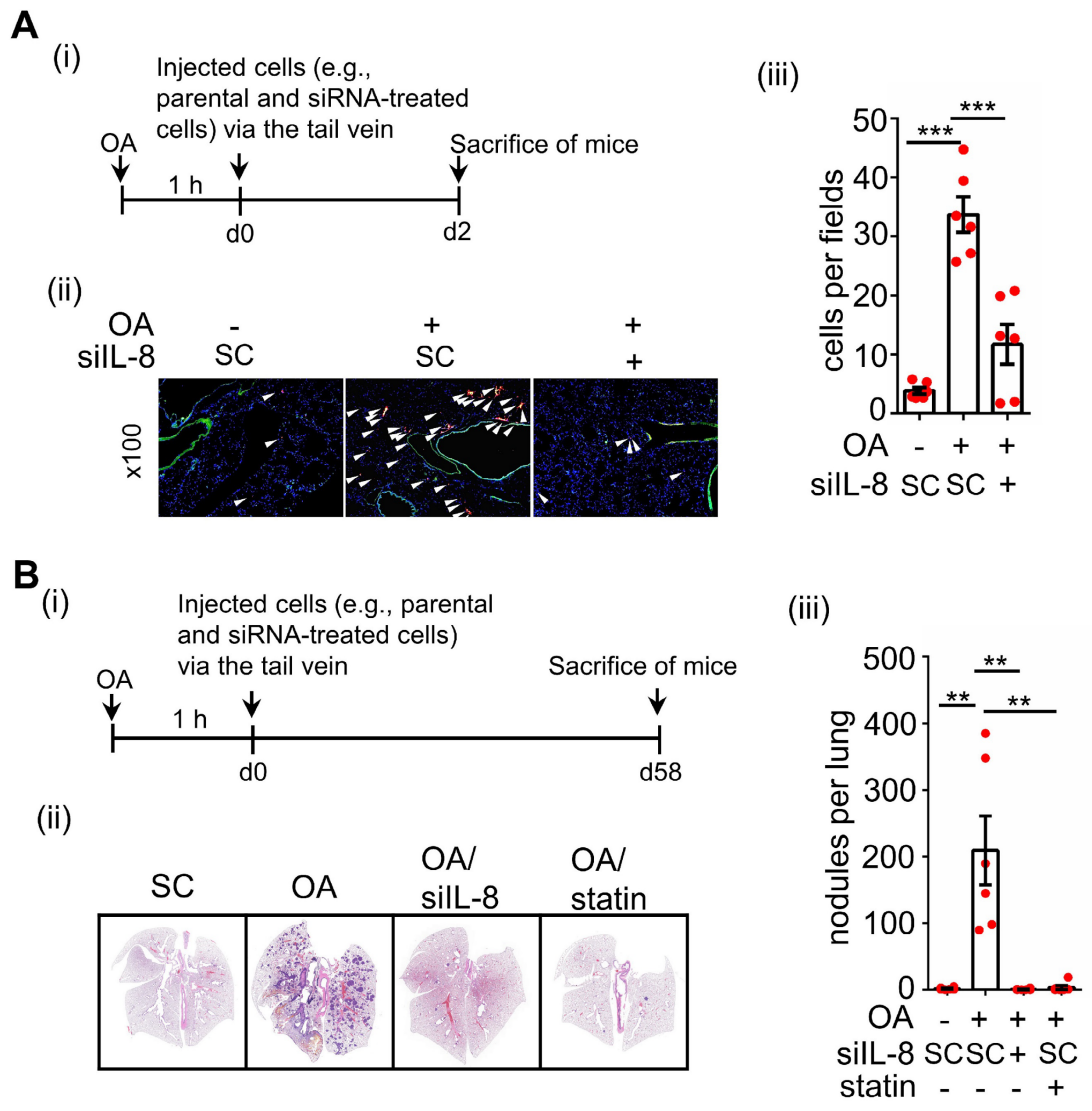
** OA-induced IL-8 is essential for the metastasis of CRC cells in mice. (A-B)** In vivo extravasation assay (A) and pulmonary metastasis assay (B) determined tumor cells penetrating pulmonary blood vessels and metastatic growth of tumor cells in the lungs, respectively. SW480 cells were transfected with 5 nM IL-8 siRNA, treated with 10 μM statin, labeled with Dil, and then injected into the tail vein of SCID-NOD male mice that had been preinjected with oleic acid (OA) (2 mg/kg). At 2 days (d2) or 58 days (d58) after injection of tumor cells, the mice were sacrificed, and metastatic tumor cells surrounding the lung tissue were examined as described in 'Materials and Methods.' (i). Tumor cell penetration was imaged using a microscope, and CRC pulmonary metastasis was examined using H&E staining. Arrows indicate extravasated cells. Original magnification, ×100; Dil-labeled tumor cells (red); CD31-labeled blood vessels (green); DAPI-labeled nuclei (blue) (ii). The amount of tumor cell extravasation was calculated by analyzing at least four sections and six fields. The number of micronodules per lung of each mouse was counted under a microscope. Dots indicate the number of mice (n = 6) (iii).

To further verify whether ANGPTL4/integrin/IL-8/NOX4 signaling regulates OA-promoted CRC metastasis, the essential role of IL-8 in regulating ANGPTL4-induced tumor cell invasion was examined. As expected, ANGPTL4-induced invasion was inhibited in cells treated with anti-IL-8 neutralizing antibodies (Figure S2). In addition, the depletion of IL-8 dramatically inhibited OA-promoted expression of EMT markers, such as MMP-1 and MMP-9 (Figure 4A), followed by a reduction in CRC cell migration and invasion (Figure 4B). The inhibition of cell migration and invasion by IL-8 siRNA was reversed in cells treated with recombinant human IL-8 protein (Figure 4B). To confirm whether the secreted ANGPTL4 and IL-8 triggered cell invasion, conditioned medium was harvested from OA-treated cells and incubated with IL-8 and ANGPTL4 neutralizing antibodies. As shown in Figure 4C, conditioned medium-promoted cell invasion was significantly inhibited by IL-8 and ANGPTL4 neutralizing antibodies. Intriguingly, the in vivo animal studies showed that the depletion of IL-8 significantly reduced OA-enhanced CRC cell extravasation and metastatic seeding of tumor cells in the lungs (Figure 5A-B). These results reveal that OA-induced IL-8 is essential for CRC metastasis.

### Activation of KRAS is essential for the OA-induced IL-8/NOX4 axis

**Figure 6:**
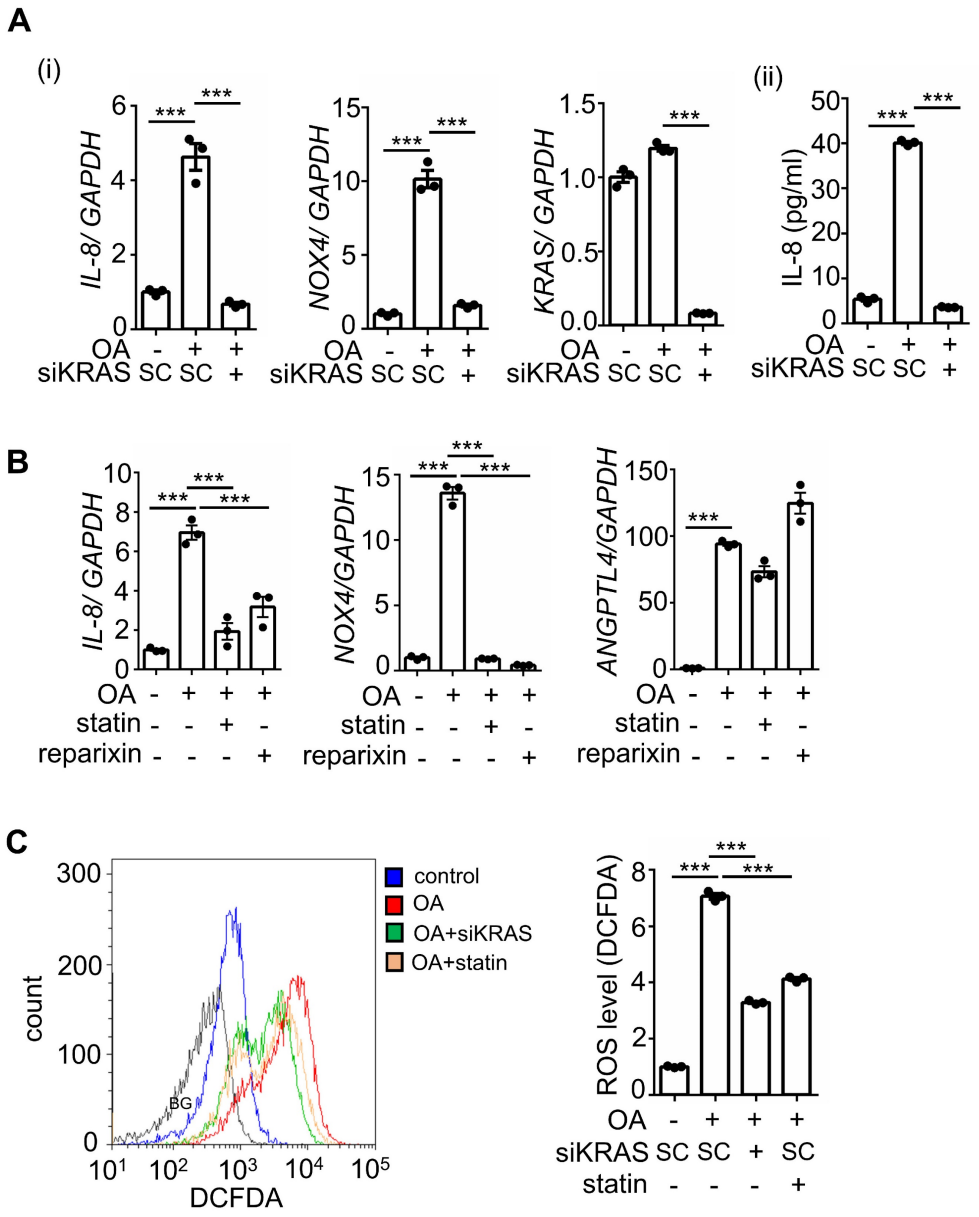
** Inhibition of KRAS and CXCR1/2 blocks the OA-induced IL-8/NOX4 axis and ROS production. (A-B)** Real-time quantitative PCR analysis was performed to examine IL-8, NOX4, KRAS, and ANGPTL4 mRNA levels in SW480 cells transfected with 5 nM KRAS siRNA or scrambled oligonucleotides (SC) (i), followed by treatment with 200 μM OA, 10 μM stain, and 10 μM reparixin for 16 h. ELISAs were performed to detect IL-8 secretion, as shown in (ii) of (A). **(C)** ROS levels were examined by flow cytometry analysis with 200 nM DCFDA staining in SW480 cells transfected with 5 nM KRAS siRNA, followed by treatment with 200 μM OA and 10 μM statin for 16 h. BG indicates background.

Previous studies have revealed that multigene mutations, including KRAS and p53, impact survival in CRC metastasis 5. To fully understand whether IL-8-regulated metastasis is associated with multigene mutations in CRC, we used CRC cell lines with mutations in oncogenes, such as KRAS and BRAF. Because KRAS mutations act in the context of cooccurring mutations, such as p53 mutations in CRC, cells containing mutants of KRAS (KRASm), p53 (p53m), and BRAF (BRAFm) were used to investigate the OA-induced IL-8/NOX4 axis. Intriguingly, OA significantly induced IL-8 expression only in cell lines containing the specific double-mutants KRASm/p53m and BRAFm/p53m, including SW480, SW620, and HT-29, but not in single mutants, non-KRASm/p53m, and non-BRAFm/p53m mutant cell lines, such as HCT-116, DLD-1, Caco2, and Colo320dm (Figure S3A). The increase in NOXs was also consistent with the induction of IL-8 in response to OA treatment in specific double-mutant CRC cell lines (Figure S3B). In addition, OA-triggered ROS production was observed in cells with specific double-mutants but not in other mutant cells (Figure S3C). These results indicate that KRASm/p53m and BRAFm/p53m play roles in OA-promoted IL-8 expression and ROS production, which may contribute to CRC metastasis. To verify whether activation of KRAS is essential for OA-induced expression of IL-8 and NOX4 and the resulting ROS production, KRAS was deleted and inhibited using KRAS siRNA and statins, respectively. As shown in Figure 6A and B, OA-induced IL-8 and NOX4 expression, but not ANGPTL4 expression, was significantly inhibited in KRAS-depleted and statin-treated cells. In addition, reparixin, an inhibitor of the IL-8 receptor CXCR1/2, also repressed the expression of IL-8 and NOX4 but not ANGPTL4 in OA-treated cells (Figure 6B). OA-induced ROS production was significantly reduced by KRAS siRNA and statin treatment (Figure 6C). These results reveal that the OA-induced IL-8/NOX4/ROS axis relies on the activation of KRAS.

### KRAS activation and oxidative stress are essential for hyperlipidemia-induced KRAS/p53-mutant CRC metastasis

**Figure 7:**
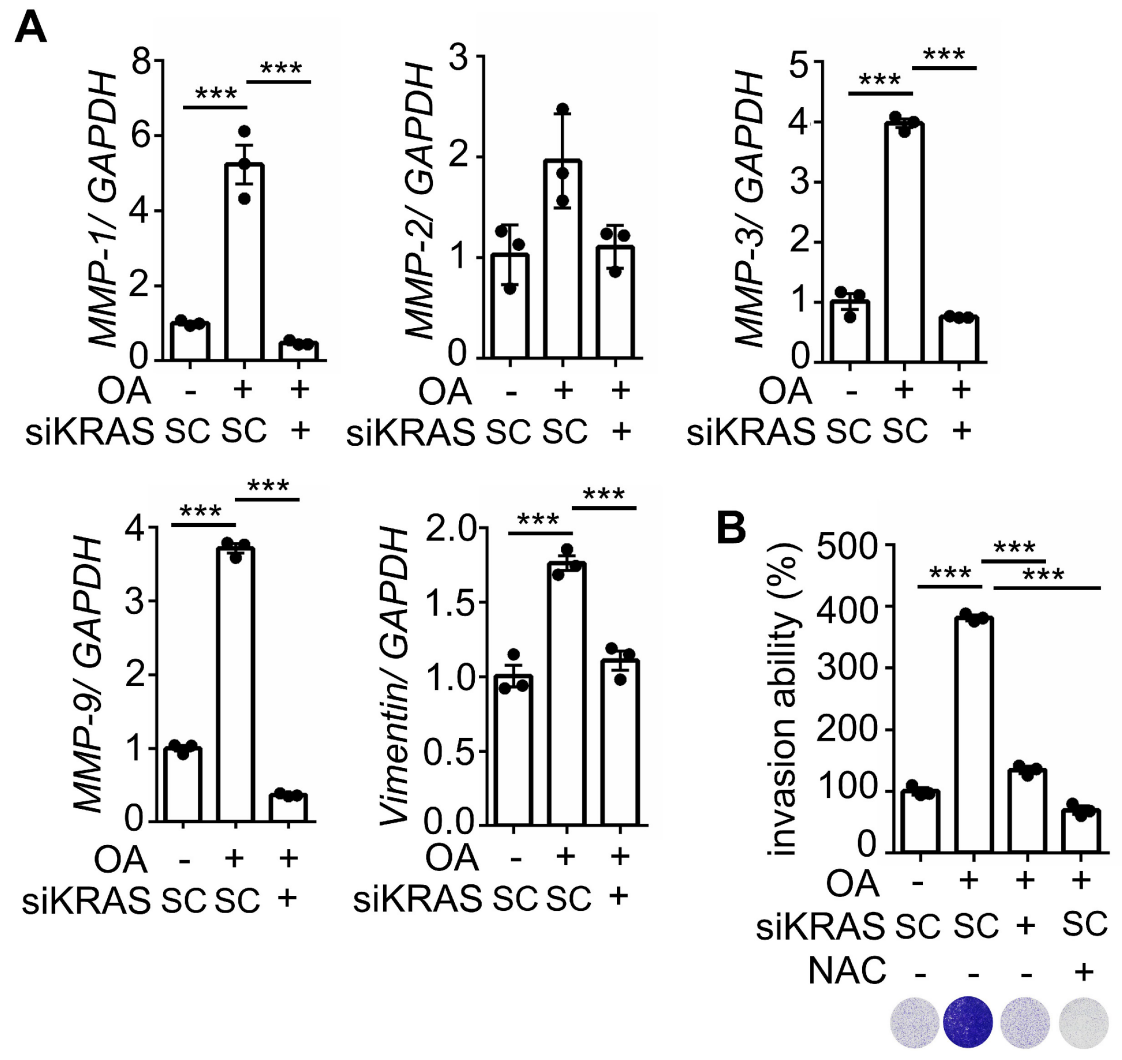
** The depletion of KRAS inhibits OA-induced EMT in CRC cells. (A)** Real-time quantitative PCR analysis was performed to examine MMP-1, MMP-2, MMP-3, MMP-9, and vimentin mRNA levels in SW480 cells transfected with 5 nM KRAS siRNA, followed by treatment with 200 μM OA for 16 h. **(B)** Invasion assays were performed using SW480 cells transfected with 5 nM KRAS siRNA and then treated with 200 μM OA and 10 mM NAC for 72 h. Invasive cells were stained with crystal violet, imaged under a microscope (lower panel), and then solubilized with 10% acetic acid. The absorbance was measured at a wavelength of 595 nm (upper panel).

**Figure 8:**
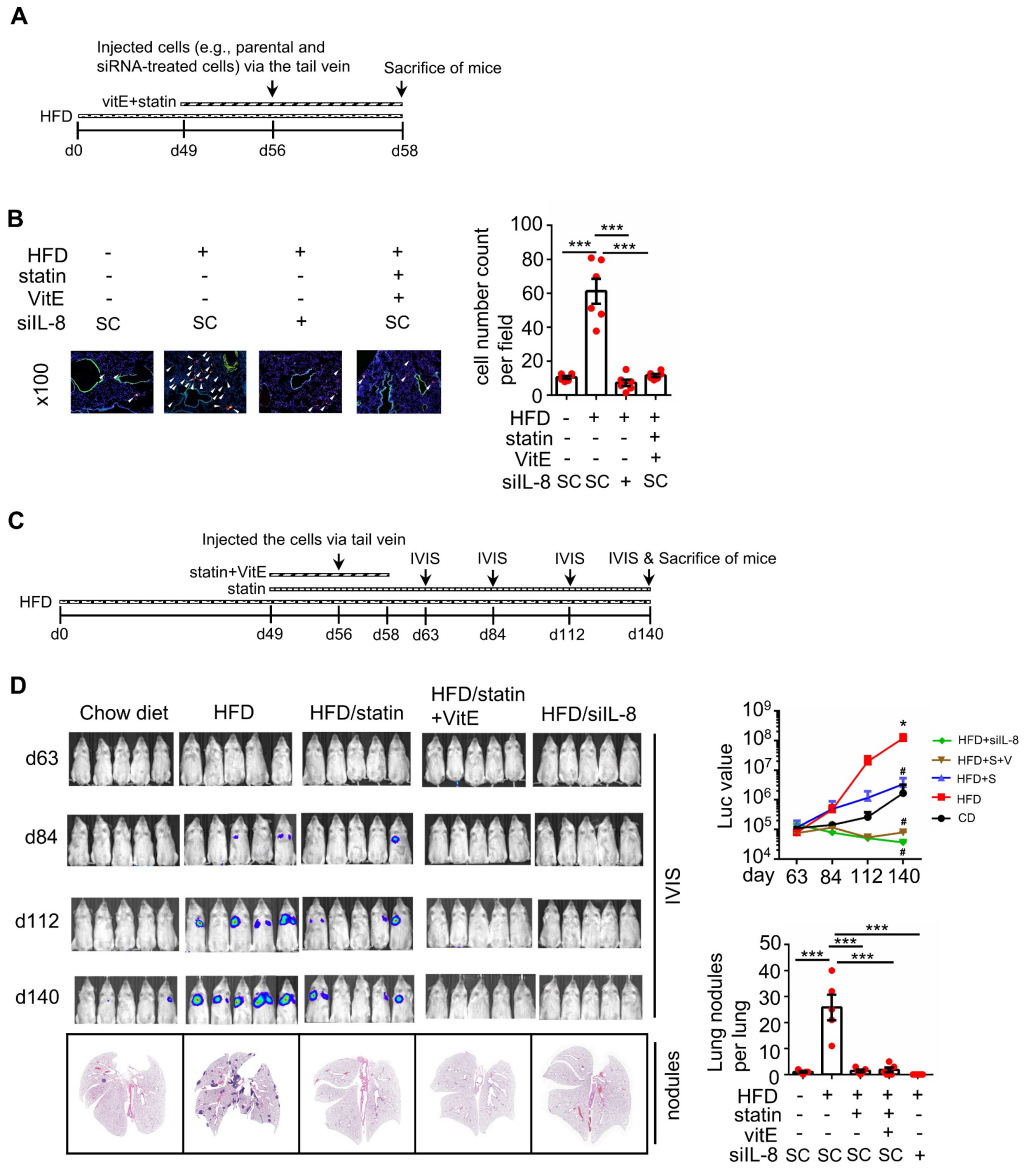
** Statin treatment, vitamin E treatment, and IL-8 depletion inhibit metastasis of CRC in high-fat diet mice. (A-B)** Tumor cells penetrate pulmonary blood vessels, which was determined by an in vivo extravasation assay. Dil-labeled SW480 cells were transfected with 5 nM IL-8 siRNA and then injected into the tail vein of high-fat diet-fed mice. As indicated, the mice were fed vitamin E (100 mg/kg) and statins (10 mg/kg). Two days after injecting tumor cells, the mice were sacrificed to examine metastatic tumor cells surrounding the lung tissue as described in 'Materials and Methods.' (A). Tumor cell penetration was imaged using a microscope (B, left panel). Arrows indicate extravasated cells. Original magnification, ×100; Dil-labeled tumor cells (red); CD31-labeled blood vessels (green); DAPI-labeled nuclei (blue). The amount of tumor cell extravasation was calculated by analyzing at least four sections and six fields (B, right panel). Dots indicate the number of mice (n = 6). **(C-D)** CRC pulmonary metastasis was assessed using SW480-Luc2 cells with or without IL-8 depletion. Cells were injected into the tail vein of high-fat diet-fed mice. As indicated, the mice were fed vitamin E (100 mg/kg) and statin (10 mg/kg) (C). After 1-12 weeks, the growth of the metastatic CRC tumor was detected using the IVIS imaging system, and then the mice were sacrificed. CRC pulmonary metastasis was examined using H&E staining (D, left panel). The luminance intensity and the number of micronodules per mouse lung were counted (D, right panel). Dots indicate the number of mice (n = 5). *: p < 0.05 HFD vs. CD; #: p < 0.05 HFD vs. HFD+S or HFD vs. HFD+ S+ V or HFD vs. HFD+ siIL-8. CD: chow diet; S: statin; V: vitE.

We further investigated whether KRASm/p53m confers OA-promoted CRC metastasis. The results showed that the inactivation of KRAS using KRAS siRNA and statins and inhibition of CXCR1/2 by reparixin dramatically inhibited the expression of OA-induced EMT markers (Figure 7A and Figure S4A), followed by a reduction in OA-promoted CRC cell invasive ability (Figure 7B and Figure S4B). In addition, depletion of ROS by NAC also significantly repressed the invasive ability of OA-treated cells (Figure 7B). These results suggest that OA-induced cell invasion occurred through activation of the KRAS/IL-8 axis. To confirm the effect of KRAS activation in regulating hyperlipidemia-associated CRC metastasis, a study of in vivo extravasation of tumor cells was performed. The mice were fed a high-fat diet (HFD) to mimetic hyperlipidemia and showed higher levels of long-chain free fatty acids (FFA) in the circulation (Figure S5A). In addition, the FFA, including OA, PA, and LA, also significantly enhanced the invasive ability of CRC cells (Figure S5B). These results suggest that HFD mice obtain higher levels of FFA in environment, which may correlate with tumor metastasis. To investigate tumor metastasis in HFD mice, parental and statin-treated CRC cells were injected into the tail vein of HFD-fed mice (Figure S5C). The results showed that HFD significantly triggered CRC extravasation, which was inhibited in statin-pretreated cells (Figure S5D), which is similar to the finding that OA-induced extravasation was inhibited by statins (Figure 5B). To further verify whether statins and antioxidants prevent CRC metastasis in mice with hyperlipidemia, mice were fed statins and vitamin E before injection of CRC cells into the tail vein (Figure 8A). First, the depletion of IL-8 inhibited HFD-triggered extravasation, confirming the essential role of IL-8 in hyperlipidemia-associated CRC metastasis (Figure 8B). It is worth noting that the combination of a statin with vitamin E dramatically inhibited HFD-promoted CRC extravasation (Figure 8B). To investigate further whether statins and antioxidants also inhibit the formation of metastatic lung nodules of CRC, the mice were orally administered long-term treatment of a statin or short-term treatment of the combination of vitamin E and a statin, as shown in Figure 8C. The results showed that HFD-triggered lung metastasis was dramatically inhibited in mice fed a statin and the combination of vitamin E and a statin (Figure 8D). In addition, the knockdown of IL-8 in cells significantly inhibited lung metastasis (Figure 8D). These results reveal that hyperlipidemia-induced KRASm/p53m CRC metastasis relies on the activation of the KRAS/IL-8/ROS signaling pathway.

### OA-induced CRC metastasis is independent of EGFR activation

It is well known that activation of KRAS is regulated by EGFR signaling in cancer cells. In addition, the activation of EGFR signaling is important for regulating CRC progression 23. To dissect the role of EGFR in OA-induced KRASm/p53m CRC metastasis, the EGFR/KRAS downstream signaling pathways, such as ERK and AKT, were examined in EGF-treated and EGFR TKI-treated cells. As shown in Figure S6A, fatty acids, including OA, PA, and LA, had a minor effect on EGFR phosphorylation but significantly induced ERK activation. OA and EGF triggered the activation of EGFR, AKT, and ERK (Figure S6B). However, EGFR TKI and EGFR siRNA dramatically attenuated EGF- but not OA-induced ERK phosphorylation (Figure S6B-C). Intriguingly, KRAS siRNA and statin treatment significantly inhibited OA- but not EGF-triggered ERK phosphorylation (Figure S6D). These results indicate that OA-induced ERK phosphorylation relied on the activation of KRAS but not EGFR. In addition, EGF-activated ERK phosphorylation was independent of KRAS in KRASm/p53m CRC cells.

On the other hand, EGF did not affect the expression of ANGPTL4, IL-8, or NOX4, consistent with the lack of changes in OA-induced IL-8 and NOX4 expression in EGFR-depleted cells (Figure S7A). As expected, EGF also did not affect ROS production (Figure S7B). However, antioxidants, but not cetuximab, significantly inhibited ROS production even when it was triggered by the combination of EGF and OA (Figure S7B). Furthermore, inhibition of ERK using U0126 partially blocked OA-induced ANGPTL4 expression but dramatically inhibited IL-8 and NOX4 expression, followed by the inhibition of tumor cell invasion (Figure S7C). These results indicate that OA-induced expression of the IL-8/NOX4 axis was dependent on ERK but not EGFR activation. Next, we pursued why IL-8 expression was triggered by the OA-activated KRAS/ERK axis but not the EGF-activated KRAS pathway or ERK pathway. Previous studies showed that the inhibition of COX-2 reduced C/EBP homologous protein (CHOP)-promoted transcriptional activation of IL-8 in cystic fibrosis cells 24. We hypothesize that OA but not EGF could activate CHOP, resulting in the induction of IL-8 expression. As shown in Figure S8A, OA but not EGF significantly induced COX-2 gene expression. OA-induced IL-8 expression was also inhibited by the depletion of COX-2 (Figure S8A). Intriguingly, OA but not EGF significantly induced CHOP phosphorylation, which was inhibited by COX-2 knockdown (Figure S8B). The depletion of COX-2 did not affect OA-induced ERK phosphorylation or KRAS activity (Figure S8B-C). However, the depletion of ANGPTL4 and inhibition of integrin signaling significantly suppressed OA-promoted KARS activity (Figure S8C). These results suggest that the lack of response of IL-8 to EGF treatment was due to the reduced activation of CHOP. In addition, OA-induced COX2 signaling regulated the phosphorylation of CHOP but not ERK. Altogether, OA-induced IL-8 expression relies on the activation of the ANGPTL4/COX2/CHOP axis and the ANGPTL4/KRAS/ERK axis.

**Figure 9:**
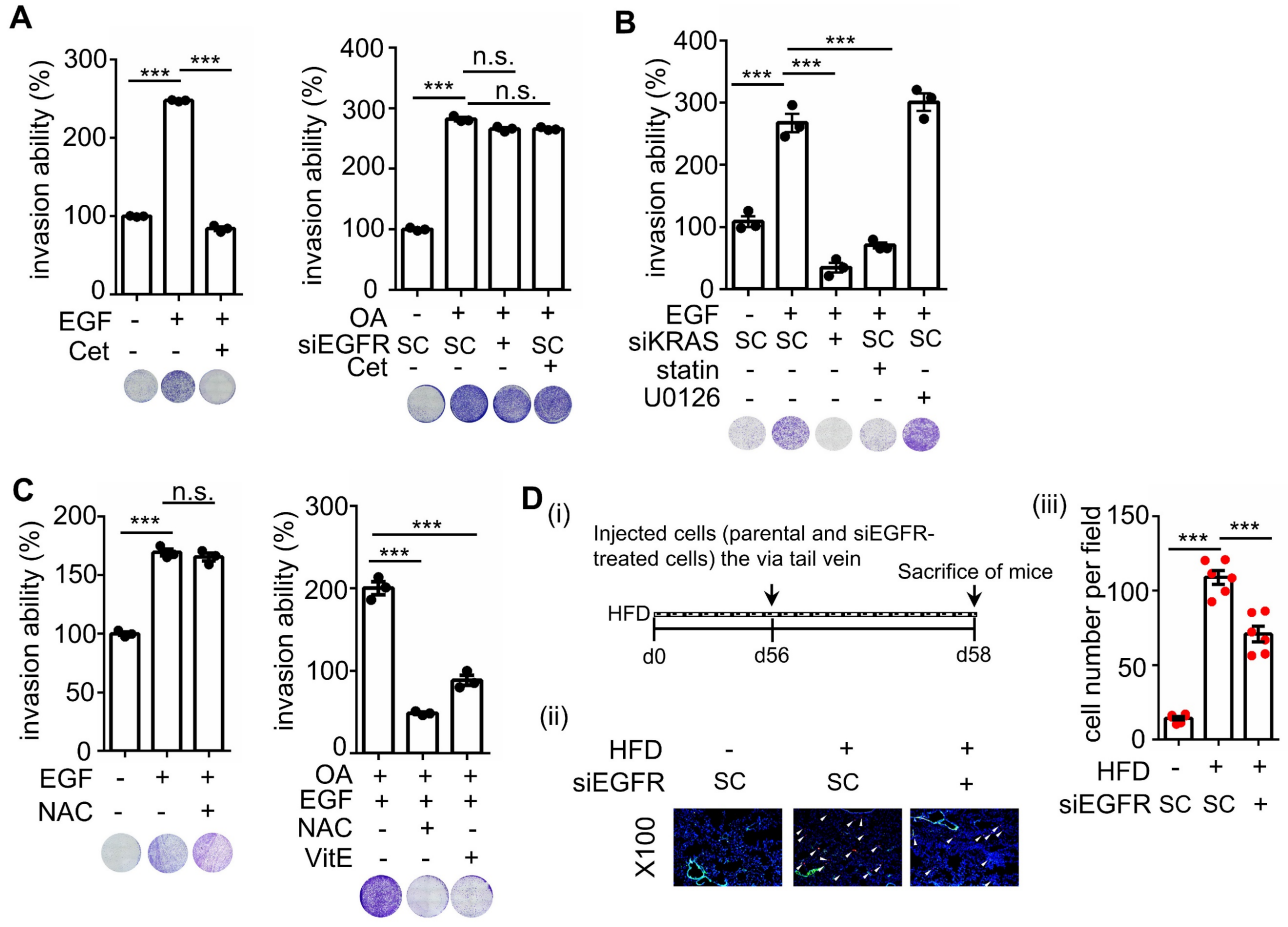
** EGF but not OA enhances invasive ability through the EGFR/KRAS pathway, not the EGFR/ERK pathway. (A-C)** Invasion assays were performed using SW480 cells transfected with 5 nM siRNA (EGFR and KRAS), followed by treatment with 200 μM OA, 50 ng/mL EGF, 30 nM cetuximab (Cet), 15 μM vitamin E (VitE), 10 mM NAC, 10 μM stain, and 10 μM U0126 for 72 h. Invading cells were stained with crystal violet, imaged under a microscope (lower panel), and then solubilized with 10% acetic acid. The absorbance was measured at a wavelength of 595 nm (upper panel). **(D)** Tumor cells penetrated pulmonary blood vessels as determined by an in vivo extravasation assay. Dil-labeled SW480 cells were transfected with 5 nM EGFR siRNA and then injected into the tail vein of high-fat diet-fed mice (HFD). Two days after injecting tumor cells, the mice were sacrificed to examine metastatic tumor cells surrounding the lung tissue as described in 'Materials and Methods' (i). Tumor cell penetration was imaged using a microscope (ii). Arrows indicate extravasated cells. Original magnification, ×100; Dil-labeled tumor cells (red); CD31-labeled blood vessels (green); DAPI-labeled nuclei (blue). The amount of tumor cell extravasation was calculated by analyzing at least four sections and six fields. Dots indicate the number of mice (n = 6) (iii).

To reconfirm whether OA-induced CRC metastasis is independent of EGFR activation, the invasive ability of cetuximab-treated cells was examined. As shown in Figure 9A, cetuximab significantly inhibited EGF- but not OA-promoted cell invasion. Notably, siKRAS and statins but not U0126 inhibited EGF-induced cell invasive ability (Figure 9B), indicating that EGF-triggered cell invasion occurred through KRAS but not ERK activation. In addition, the antioxidants did not affect EGF-triggered cell invasion (Figure 9C). Nevertheless, they inhibited the invasive ability of CRC cells triggered by the combination of OA and EGF (Figure 9C). Based on these results, we examined the effect of EGFR inhibition on tumor extravasation. We found that the depletion of EGFR partially suppressed HFD-triggered extravasation of cells (Figure 9D). These results indicate that OA-induced CRC metastasis occurred through KRAS/ROS but not EGFR activation. EGF-promoted CRC invasion relied on KRAS but not the ERK pathway, which may also partially contribute to HFD-associated CRC extravasation.

## Discussion

**Figure 10:**
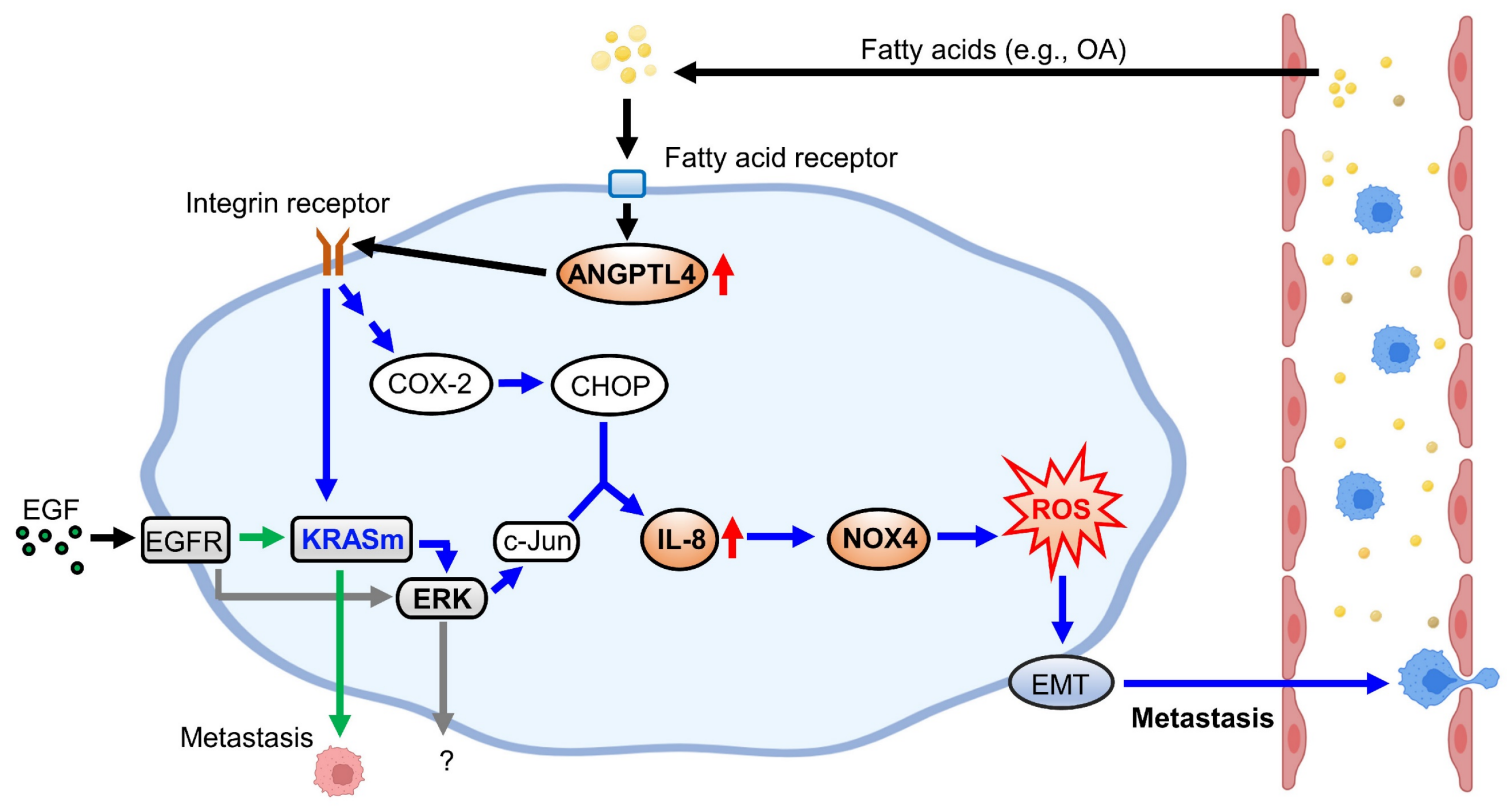
** Schematic diagram of molecular mechanisms regulating EGF- and OA-induced metastasis in KRAS/p53-mutant CRC.** Environmental EGF and fatty acids promote the metastatic properties of KRAS/p53 mutant CRC cells. The OA-induced IL-8/NOX4 axis relies on activation of the ANGPTL4/COX-2/CHOP and ANGPTL4/KRAS/ERK pathways. However, EGF-triggered cell metastasis depends on the EGFR/KRAS but not the EGFR/ERK pathway.

KRAS/p53-mutant CRC, compared with CRC with mutations in one or none of these genes, acquires high metastatic ability and anticancer drug resistance, such as cetuximab resistance, which confers poor outcomes in patients 5. Therefore, understanding the molecular mechanisms regulating KRAS/p53-mutant CRC metastasis is required to improve the treatment of cetuximab-resistant CRC metastasis. As presented in Figure 10, this study showed that the activation of the ANGPTL4/IL-8/NOX4 axis and KRAS is essential for hyperlipidemia-promoted metastasis. Although the activation of KRAS is required for CRC metastasis, the ANGPTL4/IL-8/NOX4 axis plays a unique role in regulating OA- but not EGF-enhanced metastasis of KRAS/p53-mutant CRC. These results suggest that metabolic disorders, such as hyperlipidemia, may impact the treatment of metastatic tumors such as CRC. We also showed for the first time that combining antioxidants and inhibition of KRAS can improve therapeutic efficacy in the metastasis of cetuximab-resistant CRC with hyperlipidemia status. In addition, IL-8 levels were more prominent in OA treatment than EGF treatment, suggesting that IL-8 can be a diagnostic or prognostic marker for predicting metastasis in CRC harboring KRAS/p53 mutations. In addition, this study reveals that the expression of ANGPTL4 contributes to the activation of KRAS in OA-promoted CRC metastasis. Other reports also show that ANGPTL4 has been linked to tumor progression via directly promoting tumor cell metastasis or modification of vasculature. For example, ANGPTL4 has been associated with modulating vascular junction and obesity-induced angiogenesis to promote tumor progression 25, 26. Our previous studies also showed that the expression of ANGPTL4 is essential for OA- and EGF-promoted tumor metastasis 12, 27. These results suggest that ANGPTL4 modulates tumor progression in response to tumor microenvironments, including hyperlipidemia.

Previous reports have shown that IL-8 overexpression relies on oncogenic gene mutations, such as KRAS mutation, in non-small cell lung cancer 28. Several factors, including TNFα, ROS, IL-1β, steroid hormones, hypoxia, and chemotherapeutic drugs, also regulate IL-8 signaling 29, 30. Specifically, IL-8 contributes to pathophysiology within the gastrointestinal tract exposed to *Helicobacter pylori* and immune factors associated with chronic inflammatory bowel disease (IBD) and gastric and colonic carcinomas 31, 32. In addition, overexpression of IL-8 in in vitro colonic epithelial cells promoted the migration and invasive ability of cells 33. Knockdown of CXCR1/2 inhibited the migratory capability of gastric epithelial cells 34. These results suggest that the upregulation of IL-8 is associated with cancer metastasis in the gastrointestinal tract. However, the effect of changes in molecular mechanisms and hyperlipidemia on the expression of IL-8 in CRC metastasis remains unclear. In this study, for the first time, we found that extracellular stimulators, such as fatty acids, impact IL-8 expression in cancer cells. We also showed that the ANGPTL4/COX-2/CHOP axis and KRAS/ERK pathway regulate fatty acid-induced IL-8 expression to promote CRC metastasis. Recently, a report showed that exosomes transfer mutant KRAS from CRC to recipient cells, such as neutrophils, to increase IL-8 expression and lead to the deterioration of CRC 35. However, the specific CRC cell types in which KRAS mutation activates IL-8 remain to be further explored. Our study revealed upregulation of IL-8 expression in CRC cell lines containing KRAS/p53 mutations in the hyperlipidemic microenvironment. These results indicate that lipid metabolism causes IL-8 upregulation to enhance CRC metastasis through KRAS signaling. It would be interesting to pursue whether hyperlipidemia induces the formation of exosome-containing mutant KRAS to regulate IL-8 expression, CRC metastasis, and the recruitment of macrophages, and this must be investigated further. In addition, we found that OA enhanced the expression and phosphorylation of EGFR in KRAS/p53-mutant CRC cells, similar to a previous report of exogenous IL-8 induction of the expression of EGFR in gastric epithelial cells 36. However, EGFR inhibition did not affect the OA-activated ANGPTL4/IL-8/NOX4 axis. These results suggest that the link between IL-8 and oxidative stress is the main cause of CRC metastasis in hyperlipidemia despite the presence of EGFR signaling activation.

Recent studies have shown the interplay between fatty acids and KRAS mutant cancers. For example, inhibiting the KRAS-HSL axis lowers lipid storage in lipid droplets, reducing the invasive capacity of KRAS-mutant pancreatic cancer 37. A high cholesterol:high-density lipoprotein (chol:HDL) ratio positively correlates with KRAS mutation in a subset of metastatic CRC 11. Although KRAS mutations or fatty acids have been correlated with ROS production to modulate cancer progression, the role of ROS in KRAS- and fatty acid-driven CRC cells has remained elusive. Our study not only showed that OA promotes KRAS activation to trigger CRC metastasis but also that the increase in ROS is essential for the induction of metastasis. These results indicate that KRAS mutants and the reciprocal effect of lipids on KRAS activation can manipulate metabolic reprogramming and oxidative stress to enhance cancer metastasis. In addition, a recent report showed that fatty acids and oxidative stress could either promote or sensitize cells to ferroptosis in RAS-driven cancers 38. Fatty acid metabolism genes are increased in cancers treated with neoadjuvant therapy, suggesting that lipid metabolism is associated with therapy resistance 39. However, the effect of lipid and oxidative stress on anticancer drug resistance in KRAS-driven CRC remains unclear. Our results showed that the OA-activated ANGPTL4/IL-8/NOX4 axis and KRAS pathway impact CRC metastasis. In spite, it is well-known that KRAS mutation impacts the survival of CRC patients. For example, KRAS mutation and activation are associated with poorer recurrence-free survival and progression-free survival in metastatic CRC 40, 41. The KRAS codon 12 mutation has been significantly linked with poorer overall survival of metastatic CRC and increased risk of recurrence and death in Duke C patients 42, 43. According to previous reports and our study, we predict that OA-activated IL-8/ROS and KRAS pathways may affect chemotherapeutic efficacy. These issues will be further investigated in KRAS/p53-mutant CRC cells.

The regulation of oxidative stress in KRAS-driven cancers has been important in fully understanding KRAS-dependent cancer progression. For example, oncogenic RAS increases superoxide production by upregulating NOX1 transcription through the MAPK pathway to promote tumor formation 44. A previous study showed the therapeutic use of vitamin C in CRC with KRAS or BRAF mutations 45. Consistent with these results, our study also showed that short-term treatment with a statin and vitamin E dramatically inhibited CRC extravasation and lung metastasis growth in HFD-fed mice. These results reveal that ROS production caused by activation of KRAS mainly confers CRC metastasis. Although hyperlipidemia, antioxidants, and inhibition of KRAS have the possibility to impact tumor metastasis through vascular modification, such as tubular formation and permeability, we conclude that hyperlipidemia can directly confer the metastatic ability to CRC cells and can be inhibited by antioxidants and statin. In addition, EGFR is a valuable target in treating CRC metastasis, as cetuximab has been widely used in CRC therapy 46. However, it is well known that CRC patients with KRAS mutations lack response to anti-EGFR monoclonal antibodies 47. Our study identified the molecular mechanisms regulating the metastasis of CRC tumor cells harboring KRAS/p53 mutants under EGF- and OA-treated conditions. Although KRAS activation was essential for OA- and EGF-enhanced metastasis, ROS production was triggered by OA but not EGF. These results indicate that the failure of anti-EGFR agents in treating KRAS/p53 mutant CRC may not be caused by mutant KRAS but rather by increased ROS production. Although combining statins with cetuximab is not associated with improved progression-free survival in KRAS mutant metastatic CRC 48, our findings suggest that combining cetuximab with statins that function as KRAS inhibitors and antioxidants may improve therapeutic efficacy in KRAS/p53-mutant metastatic CRC.

In conclusion, the key finding of the present study is that activation of the ANGPTL4/IL-8/NOX4 axis and KRAS is involved in the metastasis of hyperlipidemia-associated CRC with KRAS/p53 mutations. The metastasis of cetuximab-resistant CRC responded to combined treatment with statins and antioxidants under hyperlipidemic conditions. Our findings suggest that IL-8 can be a prognostic and diagnostic marker for the metastatic recurrence of EGFR-targeting resistant CRC. The application of antioxidants and statins may provide better outcomes for treating metastatic CRC that harbors multigene mutations and anti-EGFR resistance.

## Supplementary Material

Supplementary figures and materials and methods.
