# Supplementary material for: Oleic acid-induced metastasis of KRAS/p53-mutant colorectal cancer relies on concurrent KRAS activation and IL-8 expression bypassing EGFR activation
Source: Theranostics. 2023 Aug 21;13(13):4650–66. doi: 10.7150/thno.85855 (PMC10465226; doi:10.7150/thno.85855)
Supplement: Supplementary file 1 — Supplementary figures and materials and methods. [file thnov13p4650s1.pdf]

Supplementary Information

Oleic acid-induced metastasis of KRAS/p53-mutant colorectal cancer relies on concurrent KRAS activation and IL-8 expression bypassing EGFR activation

Shen *et al*.

Figures S1-8

Supplementary Materials and Methods

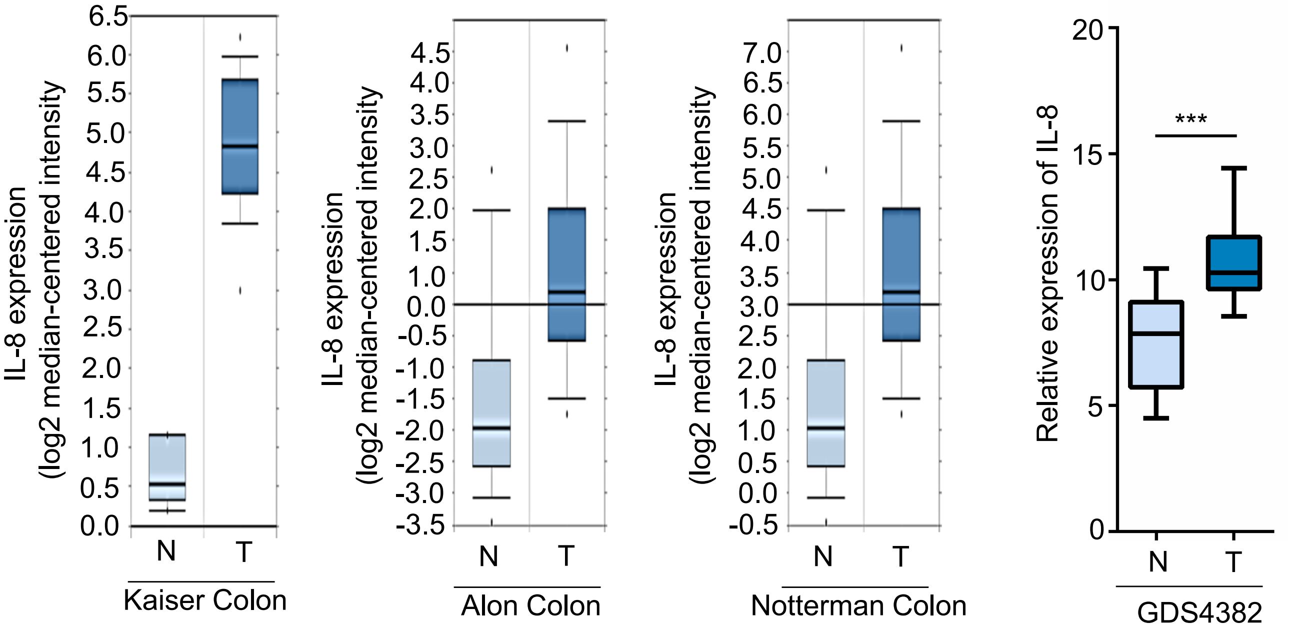

Figure S1. The expression of IL-8 mRNA in the clinical CRC dataset. IL-8 mRNA expression data in the Kaiser colon (n = 105), Alon colon (n = 84), and Notterman colon (n = 20) datasets from the Oncomine database in Oncomine (Ref: Genome biology. 2007, 8: R131; Proceedings National Academy of Sciences USA. 1999, 96: 6745-50; Experimental and Therapeutic Medicine. 2013, 6, 1499–1503) or the cancer microarray database of the NCBI website (n = 17) (GDS4382; Ref: Cancer Genomics Proteomics. 2012, 9: 67-75) were quantified.

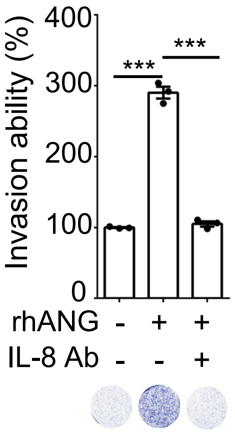

Figure S2. ANGPTL4 induces cell invasion through IL-8 expression. The invasion assay was performed in SW480 cells treated with 100 ng/mL recombinant human ANGPTL4 (rh-ANG) and 1 μg/mL anti-IL-8 antibodies for 72 h. Invading cells were stained with crystal violet, imaged under a microscope (lower panel), and then solubilized with 10% acetic acid. The absorbance was measured at a wavelength of 595 nm (upper panel).

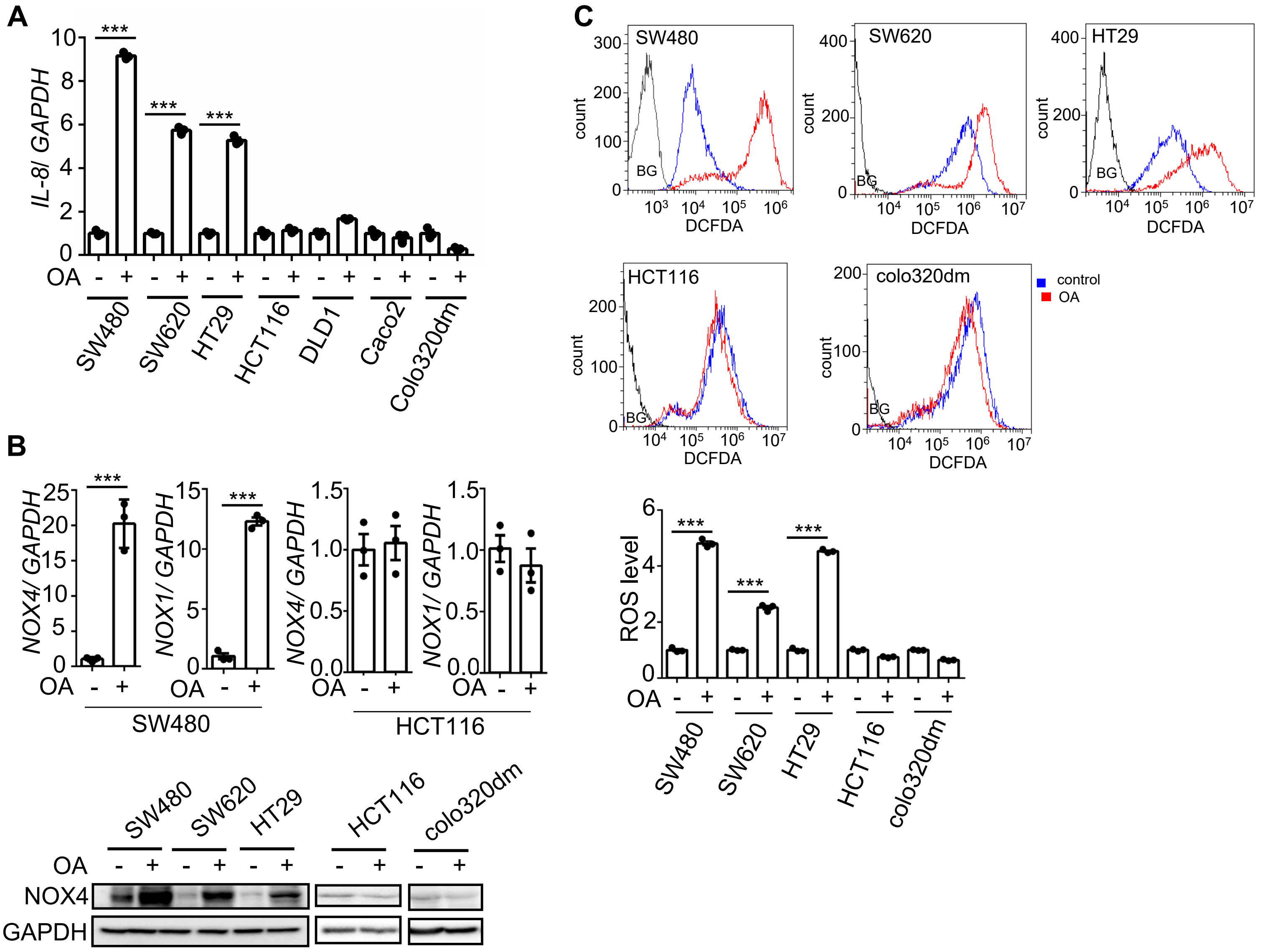

Figure S3. OA induces the expression of IL-8 and NOX4 and ROS production only in KRAS/p53-mutant CRC cell lines. (A-B) Real-time quantitative PCR analysis was used to examine IL-8, NOX-1, and NOX4 mRNA levels in various CRC cell lines treated with 200 µM OA for 16 h. Western blotting was performed using antibodies against NOX4 and GAPDH (B, lower panel). **(C)** ROS levels were estimated by flow cytometry analysis with 200 nM DCFDA staining in various CRC cell lines treated with 200 µM OA for 16 h. BG indicates background.

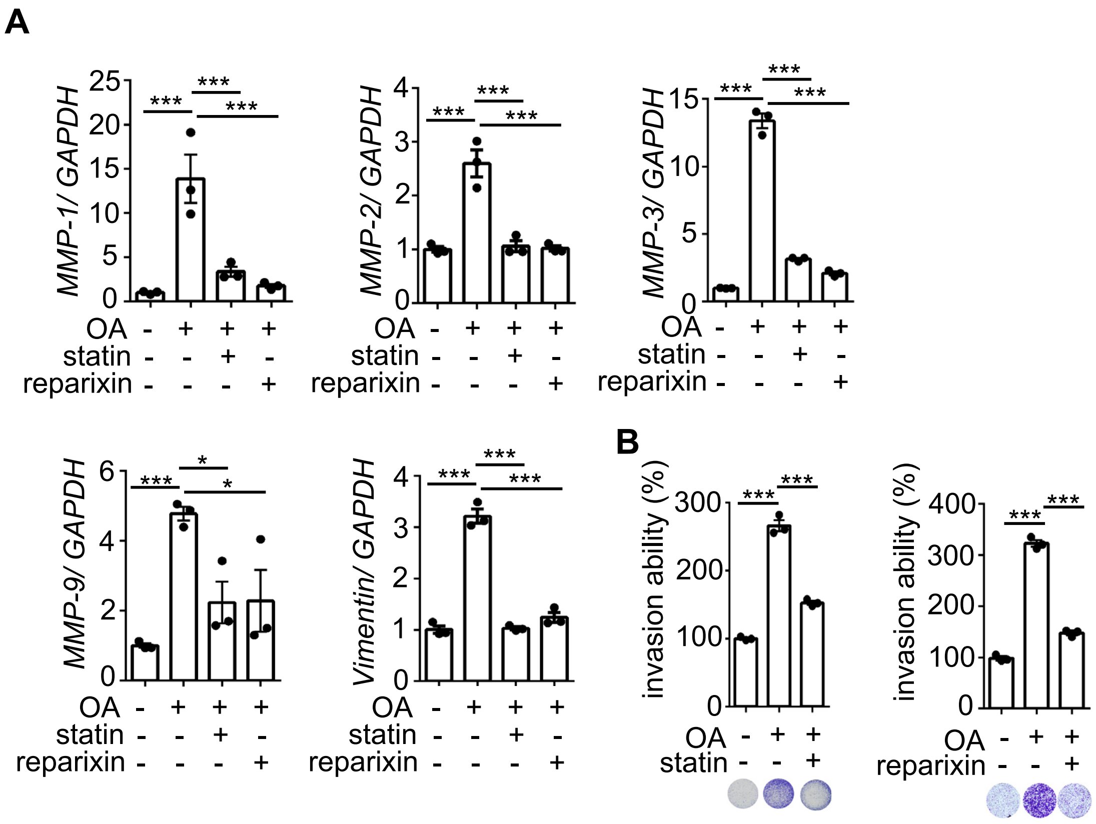

Figure S4. The inactivation of KRAS and CXCR1/2 inhibits OA-induced invasion in CRC cells. (A-B) Real-time quantitative PCR analysis (A) and invasion assays (B) were performed to examine MMP-1, MMP-2, MMP-3, MMP-9, and vimentin mRNA levels and the number of invasive cells, respectively, in SW480 cells treated with 200 µM OA, 10 µM stain, and 10 µM reparixin for 16 h or 72 h. Invasive cells were stained with crystal violet, imaged under a microscope (lower panel), and then solubilized with 10% acetic acid. The absorbance was measured at a wavelength of 595 nm (upper panel).

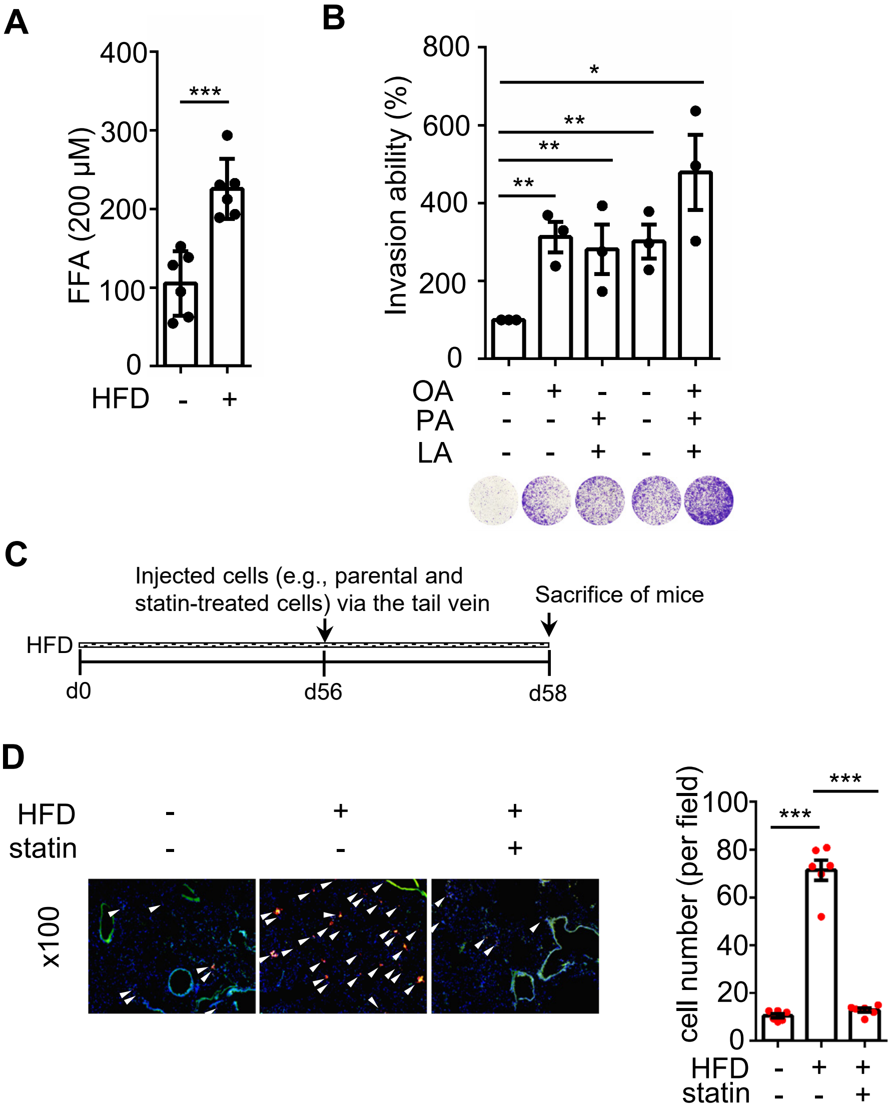

Figure S5. Statin treatment inhibits HFD-induced extravasation of CRC cells. (A) Long-chain free fatty acids (FFA) were examined in the serum of chow diet and high-fat-diet mice (HFD). Dots indicate the number of mice (n = 6). **(B)** Invasion assays were performed in SW480 cells treated with 200 µM OA, 200 µM PA, 200 µM LA, and 200 µM mixed fatty acids, including OA, PA, and LA in a ratio 1:4:1. The penetrating cells were stained with crystal violet, imaged under a microscope, and then solubilized with 10% acetic acid. The absorbance was measured at a wavelength of 595 nm. **(C-D)** Tumor cells penetrate pulmonary blood vessels, which was determined by an in vivo extravasation assay. Dil-labeled SW480 cells were pretreated with 10 µM stain for 24 h and then injected into the tail vein of high-fat diet (HFD)-fed mice. Two days after injecting tumor cells, the mice were sacrificed to examine metastatic tumor cells surrounding the lung tissue as described in 'Materials and Methods.' (C). Tumor cell penetration was imaged using a microscope (D, left panel**)**. Arrows indicate extravasated cells. Original magnification, × 100; Dil-labeled tumor cells (red); CD31-labeled blood vessels (green); DAPI-labeled nuclei (blue). The amount of tumor cell extravasation was calculated by analyzing at least four sections and six fields (D, right panel). Dots indicate the number of mice (n = 6).

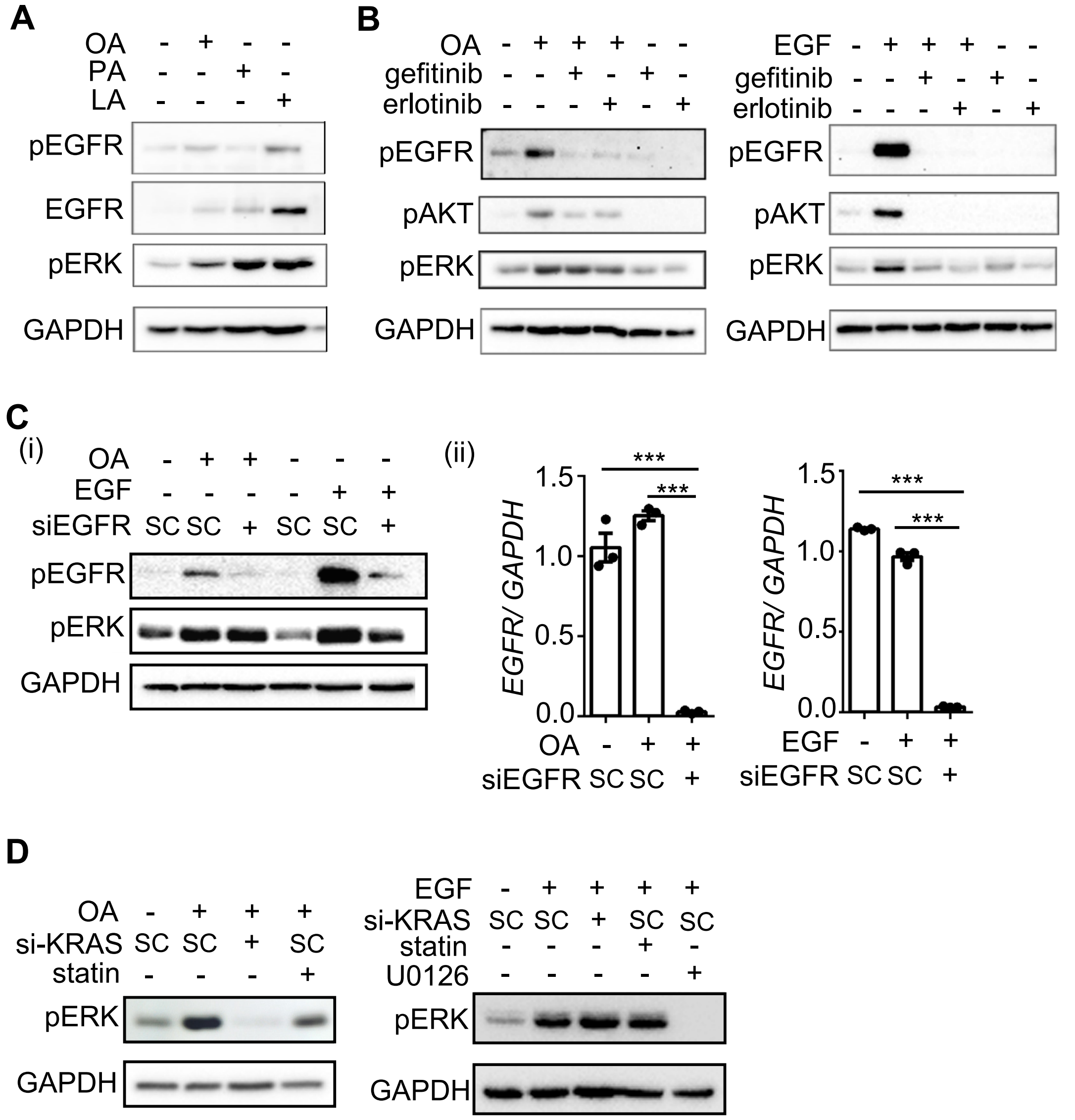

Figure S6. OA-activated ERK phosphorylation relies on KRAS but not EGFR signaling. (A-C) The expression of pEGFR, EGFR, pAKT, pERK, and GAPDH was examined using western blotting. SW480 cells were transfected with or without 5 nM EGFR siRNA and then treated with 200 μM OA, palmitic acid (PA), and linoleic acid (LA); 50 ng/mL EGF; or 10 μM gefitinib and erlotinib for 30 min as indicated. The expression of EGFR was also detected using real-time quantitative PCR in cells treated with OA and EGF for 16 h (C, ii). **(D)** Cells were transfected with 5 nM KRAS siRNA and then treated with 50 ng/mL EGF, 200 μM OA, 10 μM statin, and 10 μM U0126 for 30 min. The expression of pERK and GAPDH was examined using western blotting.

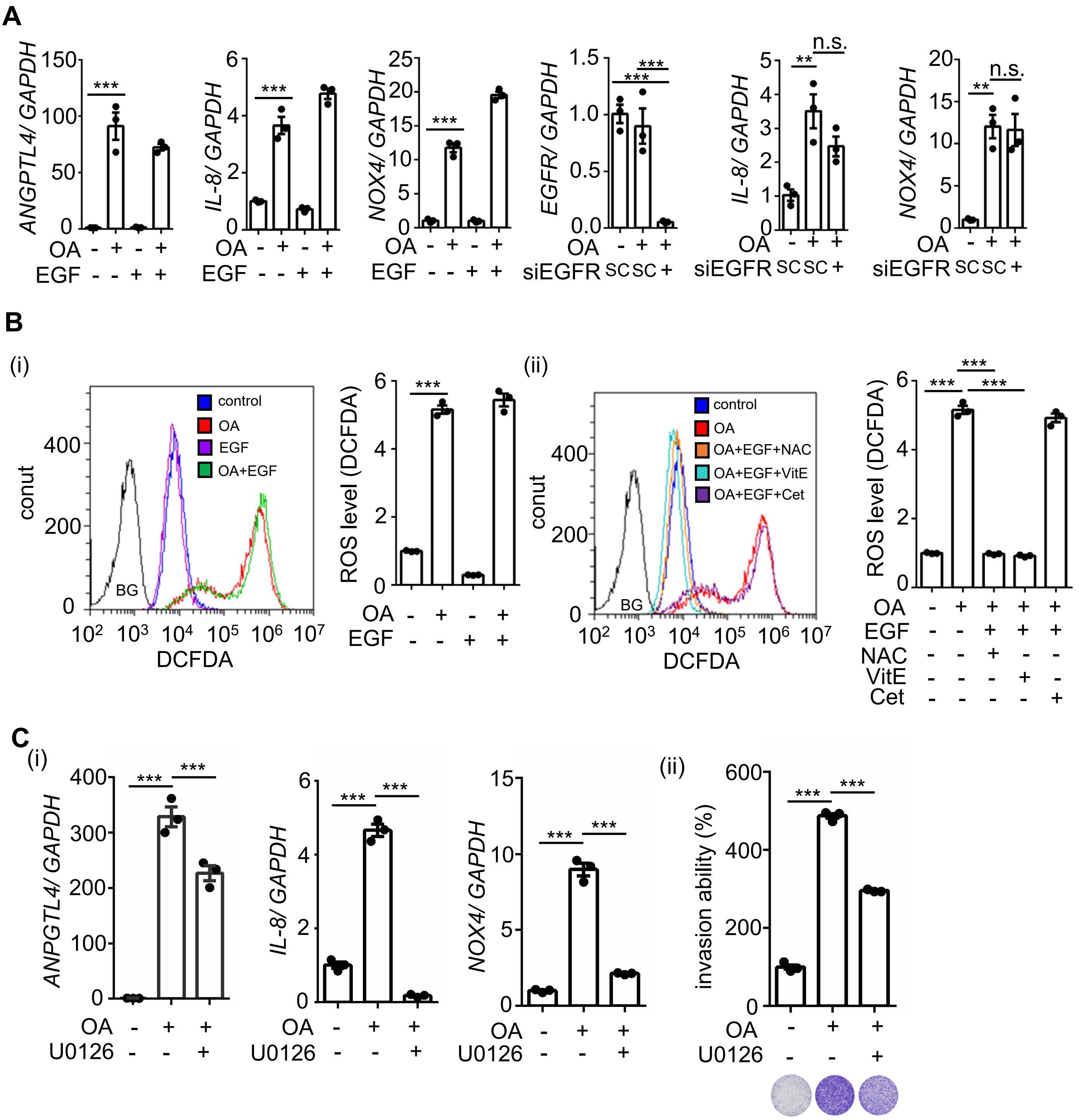

Figure S7. The OA-induced IL-8/NOX4 axis relies on ERK but not EGFR activation in CRC cells. (A) Real-time quantitative PCR analysis was performed to examine ANGPTL4, IL-8, NOX4, and EGFR mRNA levels in SW480 cells transfected with 5 nM EGFR siRNA, followed by treatment with 200 μM OA or 50 ng/mL EGF for 16 h. **(B)** The ROS levels were examined using flow cytometry analysis with 200 nM DCFDA staining in SW480 cells treated with 200 μM OA, 50 ng/mL EGF, 30 nM cetuximab (Cet), 15 μM vitamin E (VitE), and 10 mM NAC for 16 h. BG indicates background. **(C)** Real-time quantitative PCR analysis (i) and invasion assays (ii) were performed to examine ANGPTL4, IL-8, and NOX4 mRNA levels and invasive ability, respectively, using SW480 cells treated with 200 μM OA and 10 μM U0126 for 16 h and 72 h. Invading cells were stained with crystal violet, imaged under a microscope (lower panel), and then solubilized with 10% acetic acid. The absorbance was measured at a wavelength of 595 nm (upper panel).

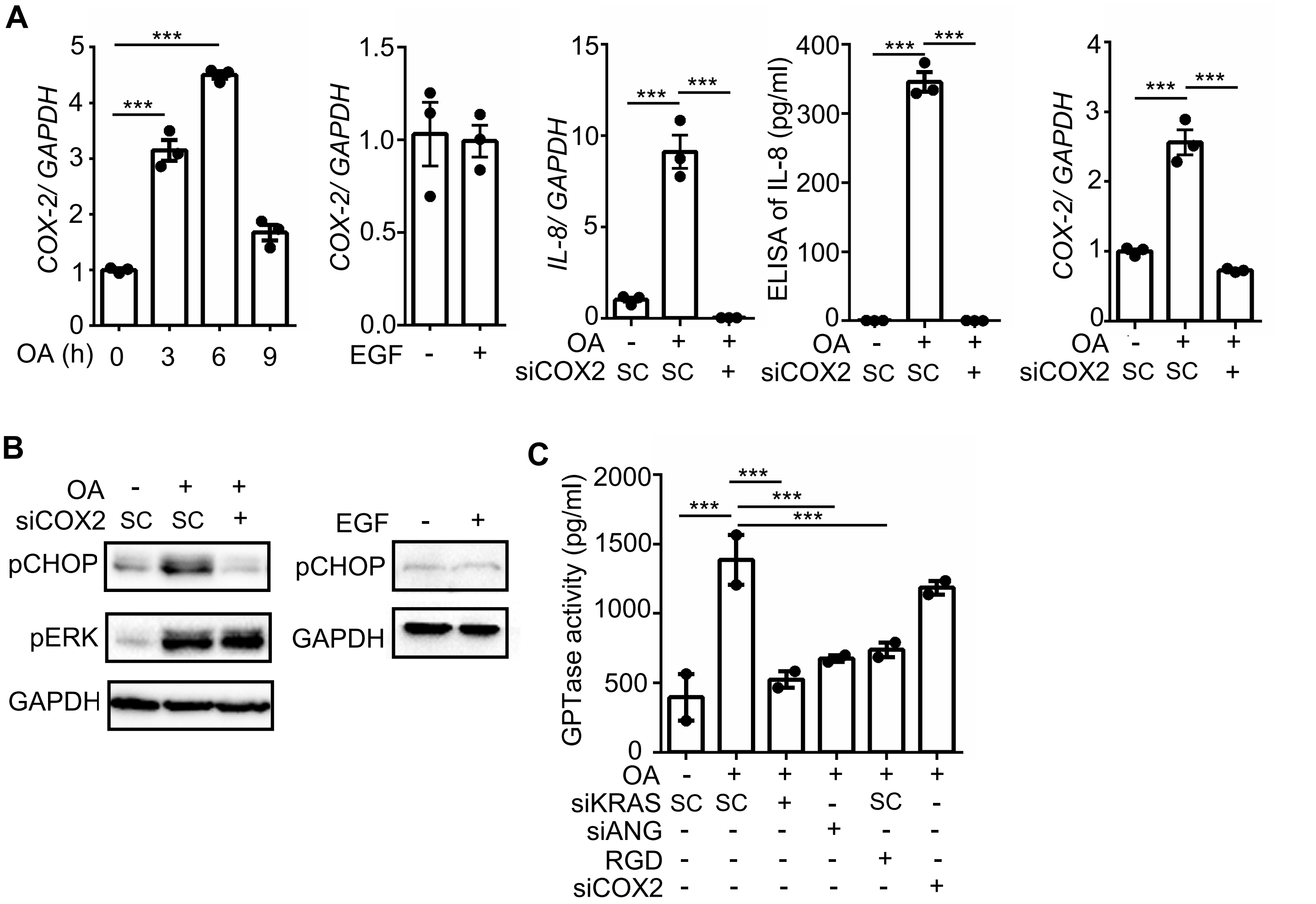

Figure S8. OA but not EGF induces the phosphorylation of CHOP through COX-2 induction in CRC cells. (A-B) Real-time quantitative PCR analysis (A) and western blotting (B) were performed to examine COX2 and IL-8 mRNA levels and the phosphorylation of CHOP and ERK, respectively, in SW480 cells transfected with 20 nM COX2 siRNA followed by treatment with 200 μM OA or 50 ng/mL EGF for 16 h or 30 min. ELISAs were performed to detect IL-8 secretion, as shown in (A). **(C)** A KRAS GTPase activity assay was performed to examine the activation of KRAS in SW480 cells transfected with 5 nM siRNA (KRAS, ANGPTL4, and COX-2), followed by treatment with 200 μM OA and 100 μM RGD for 30 min.

Supplementary Materials and Methods

Free fatty acids quantification assay

The long-chain free fatty acids were determined by using the Free Fatty Acid Quantification Assay kit (Abcam: ab65341) according to the manufacturer's instructions with some modifications. Briefly, 10 μL mice serum was collected and diluted in 90 μL assay buffer and then followed the manufacturer's instructions. The intensity of signaling (Colorimetric assay) was measured by a microplate reader at OD 570 nm after the reaction was completed.
